# Phylogeny of the genus *Yumtaax* Boucher (Coleoptera, Passalidae, Proculini): Taxonomic and evolutionary implications with descriptions of three new species

**DOI:** 10.3897/zookeys.667.10716

**Published:** 2017-04-10

**Authors:** Cristian Fernando Beza-Beza, James Beck, Pedro Reyes-Castillo, Mary Liz Jameson

**Affiliations:** 1 Department of Biological Sciences, Wichita State University, 1845 Fairmount, Box 26, Wichita, KS, USA 67260-0026; 2 Department of Biological Sciences, University of Memphis, 3774 Walker Avenue, Memphis, TN, USA 38152-3530; 3 Red de Biodiversidad y Sistemática, Instituto de Ecología, A. C., 91070, Xalapa, Veracruz, Mexico

**Keywords:** Passalidae, phylogeny, species description, *Yumtaax*

## Abstract

*Yumtaax* Boucher (Coleoptera: Passalidae) is an endemic genus from the temperate sierras of Mexico and includes six narrowly distributed species. *Yumtaax* species have been assigned to several genera of Passalidae throughout history, and a phylogenetic approach is necessary to understand species delimitation and interspecific relationships. This study reconstructed the molecular phylogeny of six *Yumtaax* morphotypes using parsimony and Bayesian analysis of DNA sequence data from the ribosomal nuclear gene region 28S and the mitochondrial gene regions 12S and cytochrome oxidase I (COI) in addition to morphological characters. Analyses recovered two well-supported *Yumtaax* clades (the *Yumtaax laticornis* and *Yumtaax imbellis* clades) that are possible sister lineages. One synapomorphic morphological character state and the geographic isolation of the group provide corroborative evidence for monophyly. Molecular phylogenetic analyses and traditional morphological examinations also resulted in the discovery of two undescribed *Yumtaax* species and the discovery of two separate evolutionary lineages (cryptic species) within *Yumtaax recticornis*. As a result we describe three new species (*Yumtaax veracrucensis* Beza-Beza, Reyes-Castillo & Jameson, **sp. n.**, *Yumtaax cameliae* Beza-Beza, Reyes-Castillo & Jameson, **sp. n.**, and *Yumtaax jimenezi* Beza-Beza, Reyes-Castillo & Jameson, **sp. n.**), redescribe two species (*Yumtaax recticornis* [Burmeister 1847] and *Yumtaax laticornis* [Truqui 1857]), and provide a key to all nine *Yumtaax* species. This study is one of two studies to use molecular data to evaluate the evolutionary relationships of a genus of Bess Beetles (Passalidae), an ecologically important insect group exhibiting low morphological variability and heretofore lacking molecular phylogenetic study.

## Introduction

*Yumtaax* Boucher (Coleoptera: Passalidae: Proculini) is an endemic genus of the southern and eastern Sierra Madre (Boucher 2006). As other members of the family Passalidae, these beetles feed on rotten wood and are important in the process of nutrient cycling in forests (Cano and Schuster 2012). Due to competition for this food resource with other Passalidae and resulting resource partitioning, *Yumtaax* species specialize on feeding in the periphery of large logs or on twigs and branches with a diameter less than 15 cm (Castillo and Reyes-Castillo 1984). Species of *Yumtaax* are associated with high altitude habitats such as cloud and pine-oak forests (Castillo and Reyes-Castillo 1984, Boucher 2006).

**Figures 1–3.**
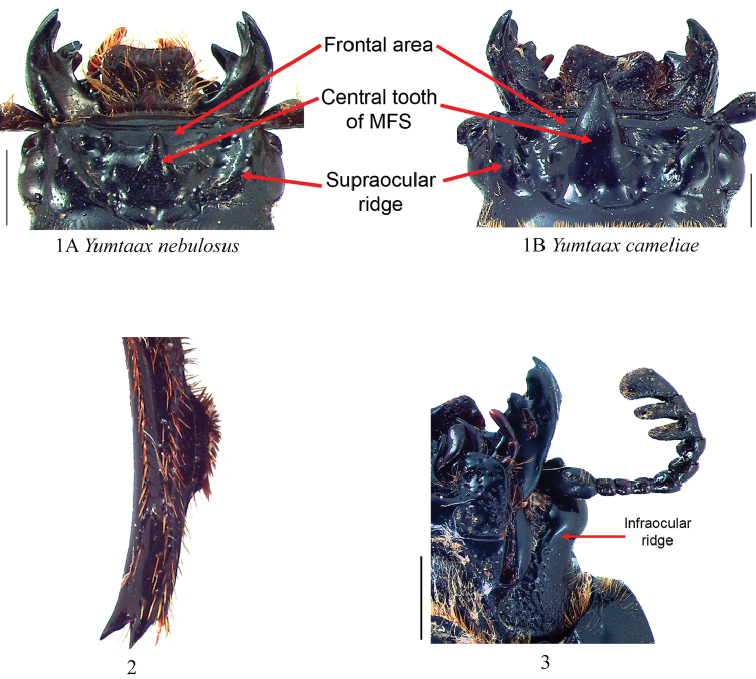
Morphological structures for *Yumtaax* species. **1** Head structures for identification of *Yumtaax* species. Central tooth of the mesofrontal structure (MSF) short (**1A**) versus long (**1B**). Eye size large in *Yumtaax nebulosus* (distal edge of the eye surpasses the distal edge of the canthus); eye size reduced in *Y. cameliae* (distal edge of the eye does not surpass the distal edge of the canthus). Scale bars: 1 mm **2** Mesotibia showing dorsal ridge elevated at the middle and setose (lateral view) of *Yumtaax cameliae* sp. n. **3** Head, ventral view, of *Yumtaax cameliae* sp. n. showing the infraocular ridge. Scale bar: 1.5 mm

*Yumtaax* was described by Boucher (2006) for six species previously considered by Castillo and Reyes-Castillo (1984) as part of the genus *Petrejoides* Kuwert: *Yumtaax recticornis* (Burmeister, 1847), *Yumtaax laticornis* (Truqui, 1857), *Yumtaax imbellis* (Casey, 1897), *Yumtaax nebulosus* (Castillo & Reyes-Castillo, 1984) (Fig. 1A), *Yumtaax mazatecus* (Castillo & Reyes-Castillo, 1984), and *Yumtaax olmecae* (Castillo & Reyes-Castillo, 1984). Boucher (2006) considered this a morphologically and biogeographically cohesive group that deserved generic status based on the dorsal mesotibial ridge that is elevated at the middle and setose on its dorsal edge (Fig. 2). *Yumtaax* species exhibit low morphological variability (Castillo and Reyes-Castillo 1984), rendering a traditional morphological phylogenetic approach of limited utility. A traditional morphological approach in combination with molecular data are needed to define species and reconstruct the phylogeny of the genus. Molecular data have historically proven useful in the family Passalidae (Villatoro 1997, Archila 2009, Beza-Beza et al. 2011, Jiménez-Ferbans et al. 2016), and these data are essential for species delimitation and phylogeny reconstruction in the absence of strong morphological data. Although passalids are a potentially informative group for understanding the dynamics of New World cloud forests (Beza-Beza et al. 2011, Schuster and Cano 2006), a strong phylogenetic hypothesis is needed for such applications. The aims of this study are to: (1) test the monophyly of *Yumtaax* and (2) reconstruct the phylogenetic relationships among *Yumtaax* species.

## Taxonomic history

Species currently considered members of *Yumtaax* have been assigned to several genera of Passalidae throughout history, and circumscription of the genus is unclear. *Yumtaax recticornis*, the type species of the genus (Boucher 2006), was originally described in *Passalus* Fabricius (Burmeister 1847) and was subsequently transferred to the passalid genera *Soranus* (Kaup, 1871), *Popilius* Kaup (Gravely, 1918), *Petrejoides* Kuwert (Reyes-Castillo, 1970), and finally *Yumtaax* (Boucher, 2006). *Yumtaax laticornis* was described by Truqui (1857) as part of *Passalus* Fabricius, but was subsequently transferred to the proculine genera *Pseudacanthus* Kaup (Kaup, 1871) and *Petrejoides* Kuwert (Reyes-Castillo, 1970). *Yumtaax imbellis* was described in the passalid genera *Soranus* (Casey, 1897) but was later transferred to the genera *Popilius* (Hincks & Dibb, 1935) and *Petrejoides* (Reyes-Castillo, 1970). The remaining three species (*Yumtaax nebulosus*, *Yumtaax olmecae*, and *Yumtaax mazatecus*) were considered part of *Petrejoides* (Castillo & Reyes-Castillo, 1984). Subsequently, the aforementioned six species were transferred to the new genus *Yumtaax* by Boucher (2006). This classification instability clearly illustrates a lack of consistent morphological circumscription for both the genera and species of Passalidae.

Using traditional morphology-based taxonomic methods, Reyes-Castillo (1970) was the first author to recognize shared characters among *Yumtaax* species, grouping *Y. imbellis*, *Y. laticornis*, and *Y. recticornis* in the genus *Petrejoides* along with *Petrejoides tenuis* Kuwert, *Petrejoides jalapensis* (Bates), and *Petrejoides orizabae* Kuwert. In a subsequent revision of *Petrejoides*, Castillo and Reyes-Castillo (1984) proposed three species groups, two of which included species currently considered *Yumtaax* (Boucher, 2006). The monotypic “laticornis species group” included *Yumtaax laticornis* and the “recticornis species group” included *Yumtaax recticornis*, *Y. imbellis*, *Y. nebulosus*, *Y. olmecae*, and *Y. mazatecus* along with *Petrejoides tenuis* (Castillo & Reyes-Castillo, 1984).

The morphological characters of the *Y. laticornis* species group include a short frontal area (Fig. 1B), dorsal mesotibial ridge elevated at the middle (described as “quilla dorsal de la tibia II corta” by Castillo and Reyes-Castillo 1984) (Fig. 2), presence of the infraocular ridge (Fig. 3), and the striatopunctatus-type mesofrontal structure (MFS) (Castillo and Reyes-Castillo 1984). The morphological characters of the *Y. recticornis* species group include the short frontal area (Fig. 1A), dorsal mesotibial ridge that is elevated at the middle (Fig. 2), and the central tooth of MFS short (Fig. 1A) (long in *P. tenuis*) (Castillo and Reyes-Castillo 1984). Morphological character states shared by *Yumtaax* species (*P. laticornis* species group + *P. recticornis* species group [- *P. tenuis*]) include the short frontal area (Fig. 1A, B) and the dorsal mesotibial ridge elevated at the middle (Boucher 2006).

### Relationships of the genus *Yumtaax*

The tumultuous nomenclatural history of *Yumtaax* species is due in part to the lack of molecular phylogenetic study of generic relationships in Passalidae. Most phylogenetic studies in the family have concentrated on the resolution of deeper relationships (subfamily and tribal level) (e.g., Fonseca 1987, Gillogly 2005, Boucher 2006, Fonseca et al. 2011) or have addressed the phylogeny of genera using morphology alone (e.g., Marshall 2000, Schuster et al. 2003, Boucher 2015, Jiménez-Ferbans and Reyes-Castillo 2015). The most complete generic-level phylogenetic analysis of Passalidae is that of Boucher (2006), who conducted a phylogenetic analysis of the tribe Proculini based on 51 morphological characters. Based on this analysis, Boucher placed *Yumtaax* within the tribe Proculini and hypothesized that *Yumtaax* is sister to *Spurius* Kaup (*Yumtaax* + *Spurius*), that the *Yumtaax* + *Spurius* clade is sister to *Popilius sensu* Boucher (2006), and that this clade (*Yumtaax* + *Spurius* + *Popilius*) is sister to *Petrejoides* sensu Boucher (2006).

## Research design and methods

### Taxon selection

To address the hypothesis that *Yumtaax* is a monophyletic group, seven operational taxonomic units (OTUs) were included. *Yumtaax recticornis* *sensu lato* (*Y. recticornis* *s. l.*)  (type species of the genus) was represented by two OTUs: *Y. recticornis* from Veracruz (*Yumtaax recticornis* VM) and *Y. recticornis* from Oaxaca (*Yumtaax recticornis* OM). The remaining OTUs were *Y. laticornis* sensu Castillo and Reyes-Castillo (*Yumtaax* LM), *Y. imbellis*, *Y. mazatecus*, and two undescribed OTUs (Suppl. material 1). Although these undescribed OTUs possessed morphological characters that place them within *Yumtaax* (dorsal mesotibial ridge elevated at the middle and setose in its dorsal edge; Fig. 2), they were morphologically distinct from the remaining OTUs. These were referred to as the *Yumtaax* “calcahualco” morphotype (*Yumtaax* CM) and the *Yumtaax* “lacortadura” morphotype (*Yumtaax* LCM). Two species of *Yumtaax* (*Y. olmecae*, and *Y. nebulosus*) were not sampled in this study. Regarding outgroup selection, a broad phylogenetic sampling of passalids was used to test the monophyly of *Yumtaax* and address sister group relationships. Exemplar species from the proculine genera *Chondrocephalus* Kuwert, *Heliscus* Zang, *Odontotaenius* Kuwert, *Oileus* Kaup, *Popilius* Kaup, *Spurius*, *Petrejoides*, *Vindex* Kaup, and *Verres* Kaup were chosen based on the phylogenetic relationships proposed by Boucher (2006) (Suppl. material 1). The genus *Passalus* (Passalinae: Passalini) was used to root all members of Proculini (Suppl. material 1).

### Specimen acquisition, DNA extraction, and amplification

Both freshly collected and museum specimens were used (Suppl. material 1). Adults and larvae were field-collected by opening rotting logs with an axe and actively searching for tunnels, adults, and larvae. Specimens were stored in 95% ethanol and kept at a cool temperature. Muscle tissue was obtained from the right hind legs of specimens. DNA was extracted using the protocol detailed in Tagliavia et al. (2011) with two modifications. In order to more fully macerate tissue, legs were ground to a fine powder using the modified reciprocating saw approach described in Alexander et al. (2007). Additionally, 240 µl of lysis buffer with detergent was used in the first step instead of 80 µl of lysis buffer per mg of ground leg.

Two mitochondrial gene regions were used: the 5' end of the small ribosomal subunit 12S rRNA and cytochrome oxidase 1 (COI). The 12S region has been shown to be useful for distinguishing clades at various taxonomic levels within Passalidae (Archila 2009, Beza-Beza 2009), and COI has been used to study relationships at species and population levels within scarabaeoids specifically (Monaghan et al. 2005) and the identification of animal species in general (International Barcode of Life Project 2009). The nuclear 28S D2 ribosomal subunit was also utilized. This region has been used in numerous Coleoptera studies (e.g., Smith et al. 2006, Monaghan et al. 2007, Wild and Maddison 2008, Ocampo et al. 2010). The 12S and 28S regions were amplified using the following primer combinations: 12S (12S 2F/SR-N-14594); 28S (28SF/28SR) (Table 1). The COI region was amplified as two segments (C1-J-1751/C1-N-2191, C1-J-2183/TL2-M3014) (Table 1). When these primer combinations failed, internal primers were used to target smaller fragments. These internal primer combinations for COI included: C1-J-1751/C1-N-2191, C1-J-2183/MaryLiz4, and C1-J-2441/TL2-M3014 (Table 1). Internal primer combinations for 28S included: 28SF/Yoshi, Charmander/Toad, Squirtle/Peach, Bulbasaur/28SR (Table 1) (Moore et al. 2015). The 12S and 28S regions were amplified with 10 µl reactions including: 1 µl 10× Klentaq (DNA Polymerase Technology, St. Louis, MO) reaction buffer (final concentration 1×), 1 µl DNTPs (0.25 µM), 1 µl each primer (1 µM each primer), 1 µl DNA template, 0.05 µl Klentaq LA polymerase, and 5 µl DI water. Ten µl COI reactions included: 1 µl 10× Klentaq reaction buffer (1x), 1 µl DNTPs (0.25 mM), 1 µl each primer (1 µM each primer), 2 µl DNA template, 0.05 µl Klentaq LA polymerase, and 4 µl DI water. Betain PCR enhancer was added at a final concentration of 1.1 M when these standard reactions failed. The following cycling parameters were used: 1) 94°C for 2 minutes, 2) 94°C for 40 seconds, 3) variable annealing temperatures (see Table 2) for 40 seconds, 68°C for various time intervals (see Table 2). Steps 2-3 were repeated for 25 cycles. All amplicons were sequenced at the University of Chicago Comprehensive Cancer Center DNA Sequencing and Genotyping Facility.

Sequence contigs were constructed using CLC Main Workbench (CLC bio, Aarhus, Denmark). If samples required amplification with internal primers (see above) sequences were assembled using Geneious R9.1 (Kearse et al. 2012). Uninterpretable sequences were cropped or discarded using the Geneious default trimming tool. Sequences were then aligned with MEGA version 5 (Tamura et al. 2011). The Muscle algorithm with default settings (Edgar 2004) was used first, followed by Clustal W (Higgins et al. 1994) alignment with default settings. Alignments were then checked by eye and manually modified if necessary. Sections of missing data were replaced with Ns.

**Table 1.** Primers used in this study. Asterisk indicates that primers were slightly modified.

Gene region	Name	Primer Sequence	Reference
12S	12S 2F	5' TACTATGTTWMGACTTATCC 3'	Kambhampati and Smith 1995*
	SR-N-14594	5' AAACTAGGATTAGATACCC 3'	Kambhampati and Smith 1995
COI	C1-J-1751	5' GGATCACCTGATATAGCATTCCC 3'	Simon et al.1994
	C1-J-2183	5' CAACATTTATTTTGATTTTTTGG 3'	Simon et al.1994
	C1-J-2441	5' CCAACAGGAATTAAAATTTTTAGATGATTAGC 3'	Simon et al.1994
	C1-N-2191	5' CCCGGTAAAATTAAAATATAAACTTC 3'	Simon et al.1994
	Mary Liz4	5' GATGAATTWGCTAAATTACTCC 3'	Moore et al. 2015
	TL2-N-3014	5' TCCAATGCACTAATCTGCCATATTA 3'	Simon et al.1994
28S	28SF	5' CCCSSGTAATTTAAGCATATTA 3'	Whiting 2001
	Yoshi	5' CGGTTTCACGTACTCTTGAAC 3'	Moore et al. 2015
	Charmander	5' GTTCAAGAGTACGTGAAACCG 3'	Moore et al. 2015
	Toad	5' CTACWGGGGGAGAAGTGCAC 3'	Moore et al. 2015
	Squirtle	5' GTGCACTTCTCCCCCWGTAG 3'	Moore et al. 2015
	Peach	5' CTAGACTCCTTGGTCCGTGTTTC 3'	Moore et al. 2015
	Bulbasaur	5' GAAACACGGACCAAGGAGTCTAG 3'	Moore et al. 2015
	28SR	5' TCGGAAGGAACCAGCTAC 3'	Whiting et al. 1997*

**Table 2.** Annealing temperatures and extension times used in this study.

Gene region	Primer combination	Annealing temperature (°C)	Extension time at 68° C (minutes: seconds)
12S	12S 2F/SR-N-14594	45	1:30
COI	C1-J-1751/C1-N-2191	47	1:30
	C1-J-2183/TL2-N-3014	44	2:30
	C1-J-2183/MaryLiz4	51	1:30
	C1-J-2441/TL2-N-3014	50	1:00
28S	28SF/28SR	52	2:30
	28SF/Yoshi	49	1:15
	Toad/Charmander	52	0:30
	Peach/Squirtle	56	1:15
	Bulbasaur/28SR	53	1:15

### Phylogenetic analysis

Phylogenetic inference using maximum parsimony and Bayesian optimality criteria was conducted for each locus independently (COI, 12S, and 28S) and the total combined dataset (COI+12S+28S). Maximum parsimony bootstrap analyses were conducted using PAUP 4.0 (Swofford 2002) and included 1000 bootstrap replicates, each involving a heuristic search with 100 random additions. Clades with bootstrap support higher than 80% were considered well supported (Baum and Smith 2013). Bayesian analyses were performed using Mr. Bayes 3.2 (Ronquist and Huelsenbeck 2003). Each analysis included 4 independent runs of one million generations, with trees sampled every 1,000 trees generations. For the COI and 28S datasets, 500,000 extra generations were run after the first one million generations until the split frequency reached less than 0.00. A concatenated alignment of the three loci was assessed with Partition Finder v.1.1.0 (Lanfear et al. 2012), which suggested treating each locus as a separate partition with the GTR+I+G model applied to the C01 and 28S partitions and the GTR+G model applied to the 12S partition. For the mitochondrial datasets (12S and COI), the genetic code Bayes function (lset code) was set to invertebrate mitochondrial (lset code=invertmt). The first 100 trees of each run were discarded the remaining 901 trees for each run of the 12S and combine dataset were then used to create a 50% majority-rule consensus tree of posterior probability values. The remaining 1,401 trees for each run of the 28S and COI dataset were then used to create a 50% majority-rule consensus tree of posterior probability values Clades with Bayesian posterior probabilities equal to or higher than 0.95 were considered well supported (Baum and Smith 2013).

### Species delimitation and species descriptions

The species status of each OTU was evaluated using two criteria. In order to be considered a species, an OTU must (1) be morphologically distinctive and (2) the molecular phylogeny must provide either evidence of its status as an evolutionary lineage or not provide contrary evidence. Species are segments of evolutionary lineages which can be diagnosed by a variety of criteria (“The General Lineage Concept”; de Queiroz 1998, Hey 2006), among them morphological distinctiveness. We view morphological distinctiveness alone as a sufficient criterion for species diagnosis, with the supporting phylogenetic data (when present) as confirmation. The taxa we diagnose represent working hypotheses and future workers should test these hypotheses with additional criteria (Carstens et al. 2013).

Type specimens for the six described *Yumtaax* species were examined in order to properly associate species names. Species descriptions used the morphological terminology of Reyes-Castillo (1970) and Castillo and Reyes-Castillo (1984) with the following modifications: total body length was measured from the anterior apex of the left mandible to the posterior apex of the left elytrum. Head width was defined as the distance between the posterior tubercles of the supraorbital ridge. Eyes were considered large if the distal edge of the eye projected beyond the distal edge of the canthus (e.g., Fig. 1A; *Y. nebulosus*), moderately reduced if the distal edge of the eye was subequal to the canthus (e.g., Figs 1B, 9C; *Y. cameliae*), and greatly reduced if the distal edge of the eye did not surpass the distal edge of the canthus (e.g., Fig. 7C; *Y. laticornis*). Using the terminology of Reyes-Castillo (1970), borders or edges of structures and sutures are described as concave (curved posteriorly), straight, or convex (curved anteriorly).

## Results

### COI data partition

The COI mitochondrial data partition consisted of 1140 aligned characters of which 450 (39%) were variable. Of the variable characters, 366 (81%) were parsimony-informative. Because parsimony and Bayesian analyses provided concordant tree topologies, bootstrap support (BS) and Bayesian posterior probabilities (PP) are shown on a Bayesian 50% majority-rule consensus tree (Suppl. material 2). The COI dataset does not support a monophyletic *Yumtaax*. However, there was support for two separate *Yumtaax* clades: a strongly supported clade comprising *Y.* CM, *Y. mazatecus*, *Y.* LM, and *Y. recticornis* VM (= “*Y. laticornis* clade”) (1.0 PP, 95 BS) and a marginally supported clade comprising *Y.* LCM, *Y. imbellis*, and *Y. recticornis* OM (= “*Y. imbellis* clade”) (0.98 PP, 55 BS). Both clades were placed in a polytomy including species from *Chondrocephalus*, *Petrejoides*, *Verres*, *Heliscus*, *Oileus*, *Odontotaenius*, and *Popilius*.

### 12S data partition

The 12S mitochondrial data partition consisted of 356 aligned characters of which 111 (31%) were variable. Of the variable characters, 82 (73%) were parsimony-informative. Because parsimony and Bayesian analyses provided concordant tree topologies, bootstrap support (BS) and Bayesian posterior probabilities (PP) are shown on a Bayesian 50% majority-rule consensus tree (Suppl. material 3). The 12S dataset does not support a monophyletic *Yumtaax*. Similar to the COI dataset, there was support for two separate *Yumtaax* clades: a strongly supported clade comprising *Y.* CM, *Y. mazatecus*, *Y.* LM, and *Y. recticornis* VM (= “*Y. laticornis* clade”) (1.0 PP, 89 BS) and a poorly supported clade comprising *Y.* LCM, *Y. imbellis*, and *Y. recticornis* OM (= “*Y. imbellis* clade”) (0.63 PP, <50 BS). Both clades are placed in a polytomy including another clade comprising species from *Chondrocephalus*, and *Petrejoides*.

### 28S nuclear data partition

The 28S data partition consisted of 1083 aligned characters of which 184 (16%) were variable. Of the variable characters, 74 (40%) were parsimony-informative. Because the parsimony and Bayesian analyses provided concordant tree topologies, bootstrap support (BS) and Bayesian posterior probabilities (PP) are shown on a Bayesian 50% majority-rule consensus tree (Suppl. material 4). Analysis of the 28S dataset did not provide support for a monophyletic *Yumtaax*. However, most *Yumtaax* OTUs (6 out of 7) were placed within a poorly supported clade (0.71 PP, <50 BS); *Y.* CM was placed sister to *P. orizabae* with maximum Bayesian and parsimony support. This relationship was also the only strongly supported conflict between the mitochondrial and nuclear 28S datasets (Suppl. material 4).

### Total combined data

**Figure 4.**
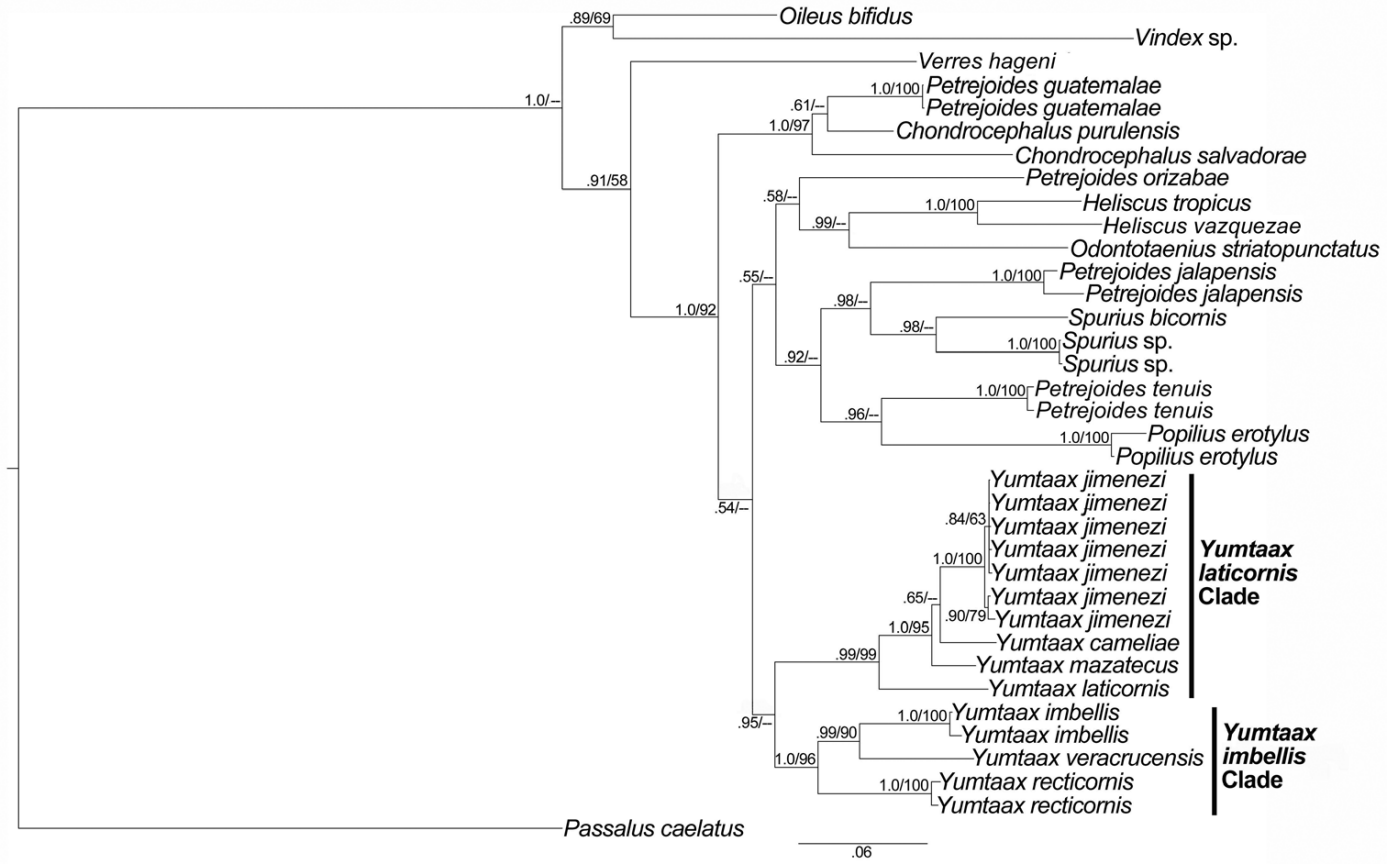
50% majority-rule consensus of Bayesian posterior probabilities resulting from analysis of the combined data. Bayesian posterior probabilities (PP) (> 0.50) and bootstrap support (BS) (> 50) are noted. *Yumtaax veracrucensis* sp. n. = *Yumtaax* LCM; *Yumtaax recticornis* = *Yumtaax recticornis* OM; *Yumtaax laticornis* = *Yumtaax* CM; *Yumtaax cameliae* sp. n. = *Yumtaax* LM; *Yumtaax jimenezi* sp. n. = *Yumtaax recticornis* VM.

The Bayesian 50% majority rule consensus phylogram resulting from analysis of the combined mitochondrial (COI, 12S) and nuclear (28S) dataset (Fig. 4) supports a monophyletic *Yumtaax* with marginal Bayesian support (0.95 PP) and no parsimony support (< 50 BS). Similar to results from the mitochondrial partitions (12S and COI, Suppl. material 2–3), there was strong support for two *Yumtaax* clades (*Y. laticornis* clade and *Y. imbellis* clade). Support for a clade comprising *Y. laticornis* (=*Y.* CM), *Y. mazatecus*, *Y. cameliae* sp. n. (=*Y.* LM), and *Y. jimenezi* sp. n. (=*Y. recticornis* VM) was strong (0.99 PP, 99 BS) (=*Y. laticornis* clade). Support for a clade comprising *Y. veracrucensis* sp. n. (=*Y.* LCM), *Y. imbellis*, and *Y. recticornis (=Y. recticornis* OM) (= *Y. imbellis* clade) was strong (1.0 PP, 96 BS). The *Yumtaax* clade was placed sister to a clade including species from *Odontotaenius*, *Petrejoides*, *Heliscus*, *Spurius*, and *Popilius*.

## Discussion

### Monophyly of *Yumtaax*

The monophyly of *Yumtaax* was supported by the total combined molecular data set examined in this study; however monophyly of *Yumtaax* was not supported when genes were analyzed individually. The 12S and COI data strongly support two *Yumtaax* clades and do not provide strong evidence for the non-monophyly of *Yumtaax*. The 28S dataset does not support a monophyletic *Yumtaax* whereas the combined data suggest a monophyletic *Yumtaax* (Fig. 4). However, a morphological character state and geographic distribution (endemic to the temperate sierras of Mexico) both suggest that *Yumtaax* species form a monophyletic group. Evaluation of all *Yumtaax* OTUs revealed that a dorsal mesotibial ridge elevated at the middle (Fig. 2) is a character state unique to this group. This contrasts with a mesotibial ridge that is elevated for the entire length of the tibia and that is observed in the remaining 16 species of this study. Boucher (2006) also considered this dorsal mesotibial ridge elevated at the middle a synapomorphy for *Yumtaax*, and thus far, the dorsal mesotibial ridge elevated at the middle has not been observed in any other species of Passalidae (approximately 930 spp.) (Boucher 2006).

A combined consideration of the molecular phylogenetic, morphological, and geographic data suggests that the best working hypothesis is of *Yumtaax* as a valid, monophyletic genus. Further study should include both broader taxon sampling (including the genera *Petrejoides* sensu Boucher, *Popilius* sensu Boucher, and the excluded species of *Yumtaax* [*Y. nebulosus*, *Y. olmecae*]) and data from additional gene regions (particularly nuclear). For the remainder of this work, *Yumtaax* is treated as a monophyletic genus.

### *Yumtaax* species delimitation

Based on combined morphological, molecular, and geographic data, we provide evidence that the *Yumtaax* OTUs analyzed in this study include seven distinctive species: *Y. imbellis*, *Y.* LCM, *Y. recticornis* OM, *Y.* CM, *Y. mazatecus*, *Y.* LM, and *Y. recticornis* VM (Fig. 4, Suppl. material 2, 3). Analysis of the combined molecular dataset recovered clear evidence for three independent evolutionary lineages (= species) corresponding to *Y. imbellis*, *Y. recticornis* OM, and *Y. recticornis* VM (Fig. 4, Suppl. materials 2, 3, 4). Although the lineage status of *Y.* LCM, *Y.* CM, *Y. mazatecus*, and *Y.* LM could not be established due to the lack of multiple samples per OTU, the phylogeny does not provide evidence that these are *not* lineages. For instance, each individual sample of these OTUs is genetically distinguishable from other *Yumtaax* lineages (has a non-zero branch length) and does not render *Y. imbellis*, *Y. recticornis* OM, or *Y. recticornis* VM paraphyletic. We treat each of these OTUs as species (see species diagnoses below).

Molecular and morphological data both suggest that *Y. recticornis* *s. l.* is composed of two independent lineages. First, eye size in *Y. recticornis* *s. l.* is geographically dependent; populations from Veracruz have reduced eyes whereas those from Oaxaca have large eyes (Castillo and Reyes-Castillo 1984). Molecular data reveal that these morphotypes form separate lineages: *Y. recticornis* VM is part of the *Y. laticornis* clade, and *Y. recticornis* OM is part of the *Y. imbellis* clade (Fig. 4). Based on examination of the type specimen of *Y. recticornis*, the name should be assigned to *Y. recticornis* OM; this species is distributed exclusively in Sierra Madre del Sur in Mexico. *Yumtaax recticornis* VM is an unnamed species that it is currently known exclusively from the transverse neo-volcanic system in Mexico. Within the *Y. imbellis* clade, molecular (Fig. 4) and morphological (see diagnosis below) data suggest that *Y.* LCM is a distinct species currently known only in the transverse neo-volcanic system in Mexico. Based on examination of type specimens in the genus *Yumtaax*, this morphotype also represents an undescribed species. Close examination of the type specimen of *Y. laticornis* indicated that this name should be applied to the *Y.* CM morphotype rather than the *Y.* LM morphotype (*Yumtaax laticornis* sensu Castillo and Reyes-Castillo 1984). Based on the type specimen, we re-circumscribe *Y. laticornis* and describe a new species for *Y.* LM.

Due to geographic isolation and morphological differences we follow Castillo and Reyes-Castillo (1984) and consider *Y. nebulosus* and *Y. olmecae* as independent, evolutionary lineages (= species) within the *Yumtaax* genus.

### Species descriptions of *Yumtaax*

As a result of this work, we describe three new species: *Y. veracrucensis* sp. n. (= *Y.* LCM), *Y. cameliae* sp. n. (= *Y.* LM), and *Y. jimenezi* sp. n. (= *Y. recticornis* VM). In order to stabilize nomenclature, we re-circunscribe two species (*Y. recticornis* and *Y. laticornis*). *Yumtaax recticornis* *s. l.* is composed of two unrelated and heretofore cryptic species (*Y. recticornis* OM and *Y. recticornis* VM). Close examination of the *Y. recticornis* lectotype and one paralectotype indicates that the name *Y. recticornis* corresponds to our *Y. recticornis* OM. Redescription of this species is necessary to re-circumscribe *Y. recticornis* sensu stricto (*Y. recticornis* *s. s.*) and distinguish it from the *Y. recticornis* VM lineage (= *Y. jimenezi*). Close examination of the *Y. laticornis* holotype indicates that this name corresponds to *Y.* CM rather than to *Y. laticornis* sensu Castillo and Reyes-Castillo (1984) (= *Y.* LM). A redescription of the holotype of *Y. laticornis* is provided to clarify this finding. As such, the genus *Yumtaax* includes nine species that can be separated morphologically using the following key.

### Key to the species of *Yumtaax*

(Modified from Castillo and Reyes-Castillo 1984, Schuster 1991)

**Table:** 

1	Apex of central tooth of mesofrontal structure (MFS) (viewed from above) extends beyond frontoclypeal suture (Fig. 1B)	**2**
1	Apex of central tooth of mesofrontal structure (MFS) (viewed from above) not reaching the frontoclypeal suture (Fig. 1A)	**4**
2	Mesofrontal structure (MFS) dorsally with scarce micro-punctures (Fig. 6C). Body length 17.5–20.0 mm. Aedeagus elongated (Fig. 6E, F). Mexican Transvolcanic Belt	***Y. veracrucensis* Beza-Beza, Reyes-Castillo & Jameson, sp. n.**
–	Mesofrontal structure (MFS) dorsally impunctate. Body length >22.0 mm. Aedeagus globose (e.g., Fig. 8F, G, H)	**3**
3	Scutellum punctate. Mesofrontal structure (MFS) of the falsus type (Fig. 7C). Distal edge of the eye not surpassing distal edge of the canthus (Fig. 7D). Brachypterous. Pico de Orizaba	***Y. laticornis* (Truqui)**
–	Scutellum impunctate. Mesofrontal structure of the striatopunctatus type (Fig. 8C). Distal edge of the eye subequal to the canthus (Fig. 8D). Macropterous. Puerto del Aire, Veracruz	***Y. cameliae* Beza-Beza, Reyes-Castillo & Jameson, sp. n.**
4	Frons with central longitudinal ridge. Southeastern Sierra Madre, Oaxaca Highlands	***Y. mazatecus* (Castillo & Reyes-Castillo)**
–	Frons without central longitudinal ridge	**5**
5	Mesofrontal structure (MFS) with dorsal groove (Fig. 9A in Castillo and Reyes-Castillo 1984). Dorsal anterior profile of elytra straight	**6**
–	Mesofrontal structure (MFS) without dorsal grove (Fig. 8A in Reyes-Castillo 1984). Dorsal anterior profile of elytra V-shaped	**7**
6	Femur I without longitudinal anterior-ventral groove. Union of elytral striae 1–10 with a row of fine punctures. Dorso-lateral surface of the pronotum punctate. Body length 16.5–19.0 mm. Eastern Sierra Madre	***Y. nebulosus* (Castillo & Reyes-Castillo)**
–	Femur I with longitudinal anterior-ventral groove. Union of elytral striae 1–10 with >1 rows of punctures. Dorso-lateral surface of the pronotum impunctate. Body length 24.0–27.0 mm. Sierra Madre del Sur, Guerrero	***Y. olmecae* (Castillo & Reyes-Castillo)**
7	Infraocular ridge absent (not as in Fig. 3). Clypeus vertical. Sierra Madre del Sur, Guerrero	***Y. imbellis* (Casey)**
–	Infraocular ridge present (Fig. 3). Clypeus inclined. Distribution Sierra Madre del sur (Sierra Juarez, Oaxaca); or Transverse neo-volcanic system (Veracruz)	**8**
8	Clypeal surface concave. Frontoclypeal suture concave. Central tooth of mesofrontal structure (MFS) largely free from frontal ridges (Fig. 5C). Distal edge of the eye projected beyond distal edge of the canthus (Fig. 5D). Aedeagus elongated (Figs 5F, G, H). Sierra Juarez, Oaxaca	***Y. recticornis* (Burmeister)**
–	Clypeal surface flat. Frontoclypeal suture straight. Central tooth of mesofrontal structure (MFS) fused with frontal ridges (Fig. 9C). Distal edge of the eye subequal to the canthus (Fig. 9D). Aedeagus globose (Fig. 9E, F, G). Transverse neo-volcanic system	***Y. jimenezi* Beza-Beza, Reyes-Castillo & Jameson, sp. n.**

#### Yumtaax recticornis

Taxon classificationAnimaliaColeopteraPassalidae

(Burmeister, 1847)

Passalus recticornis Burmeister, 1847: 508–509.
Soranus recticornis (Burmeister) [comb. n. by Kaup 1871: 105, 108].
Popilius recticornis (Burmeister) [comb. n. by Gravely 1918: 24, 26].
Petrejoides recticornis (Burmeister) [comb. n. by Reyes-Castillo 1970: 125].
Yumtaax recticornis (Burmeister) [comb. n. by Boucher 2006: 348].

##### Material examined.

105 specimens (lectotype, paralectotype, and 103 non-type specimens). *Lectotype* ♂. MEXICO: WB zoologie S. Nr. 812123. (MLU Halle). *Paralectotype* 1 ♀. MEXICO: no data (MLU Halle).

*Non-type specimens* (103 total). MEXICO: 1 ♀, Oaxaca, Amatepec (1.6 km N), alt. 1840 m, bosque mesófilo de montaña, II-28-1988 (*Reyes*, *Boucher*, *C. Castillo*). 1 ♂, Carretera Tuxtepec-Oaxaca (87 km), III-21-1967 (*R. MacGregor*); 1 ♀, (88 km), alt. 2350 m, IV-4-1986 (*A. Ibarra*); 1 ♂, 1 ♀, (119 km), X-6-1973 (*J. Mateu*); 4 ♂, 2 ♀, (153 km), alt. 2800 m, X-7-1973 (*J. Mateu*). 1 ♀, Cerro Pelón (2.8 km), V-18-1980 (*C. Castillo*, *M. L. Castillo*, *G. Quintero*, *E. Rivera*); 3 ♀, (11.4 km NE), alt. 2170 m (*P. Reyes et al.*). 1 ♀, Comaltepec, Brecha 60 (unknown locality), V-18-1980 (*C. Castillo*, *M. L. Castillo*, *G. Quintero*, *E. Rivera*); 3 ♂, 2 ♀, (4.5 km), V-18-1980 (*C. Castillo*, *M. L. Castillo*, *G. Quintero*, *E. Rivera*). 1 ♂, 4 ♀, Comaltepec, Galera San Isidro (800 m), alt. 2000 m, V-17-1980 (*C. Castillo*, *M. L. Castillo*, *G. Quintero*, *E. Rivera*); 3 ♂, 5 ♀, (3.6 km), alt. 2160 m, V-17-1980 (*C. Castillo*, *M. L. Castillo*, *G. Quintero*, *E. Rivera*). 1 ♀, La Esperanza, alt. 1670 m, V-16-1980 (*C. Castillo*, *G. Quintero*, *M. L. Castillo*, *E. Rivera*); 4 ♂, 3 ♀, (3.5 km N), alt. 1670 m, bosque mesófilo de montaña, III-1-1988 (*Reyes*, *Boucher*, *Castillo*); 1 ♀ (4 km), alt. 1800 m, V-20-1980 (*C. Castillo*, *M. L. Castillo*, *G. Quintero*, *E. Rivera*); 5 sex unknown, (6.8 km), alt. 1820 m, II-25-1984 (*P. Reyes et al.*); 1 sex unknown, (13.1 km), alt. 2030 m, II-25-1984 (*P. Reyes et al.*); 3 sex unknown, (14.1 km), alt. 1985 m, II-25-1984 (*P. Reyes et al.*); 2 sex unknown, (103.1 km), alt. 2030 m, II-25-1984 (*P. Reyes et al.*). 1 sex unknown, San Juan Lachao Viejo, km 85 de Sola de Vega (N 16°13.220' W 97°08.913), alt. 1858-1870 m, bosque mesófilo de montaña, VIII-6-2004 (*K. Araya*). 30 sex unknown, San Miguel Talea de Castro (8 km SE) (N 17°19.620' W 96°17.403), alt. 2082 m, VII-22-2007 (*O. Francke*, *H. Montaño*, *A. Valdéz*, *C. Santibañez*, *A. Ballesteros*). 3 ♂, 4 ♀, Sierra de Juárez, alt. 2000 m, VI-2-1995 (*G. Nogueira*). 2 ♂, 1 ♀, Totontepec (3.4 km S), alt. 1940 m, bosque mesófilo de montaña, II-29-1988 (*Reyes*, *Boucher*, *Castillo*). 3 ♂, 3 ♀, Valle Nacional (32 miles S), V-21/24-1971 (*H. Howden*); 1 ♀, (105-117 km E), IV-1964 (*C. R. Rotger*).

##### Diagnosis.

*Yumtaax recticornis* is a small (18.0–21.0 mm), macropterous species and is a member of the *Y. imbellis* clade (Fig. 4). This species is diagnosed by the following character combination: the clypeus is inclined (shared with *Y. jimenezi*, *Y. imbellis*, *Y. mazatecus*, *Y. nebulosus*, *Y. olmecae*; clypeus vertical in *Y. veracrucensis*, *Y. laticornis*, *Y. cameliae*), surface concave (flat in other members of *Yumtaax*), and the anterior border is concave (shared with *Y. olmecae*; flat in other members of *Yumtaax*); mesofrontal structure (MFS) of the “falsus” type (see Reyes-Castillo 1970) (shared with all members of *Yumtaax* except *Y. cameliae* that has a MFS of the “striatopunctatus” type) with the central tooth largely free (shared with *Y. veracrucensis*, *Y. laticornis*, *Y. cameliae*, *Y. mazatecus*; fused with frontal ridges in *Y. jimenezi*, *Y. imbellis*, *Y. nebulosus*, *Y. olmecae*), central tooth directed dorsally (shared with *Y. imbellis*, *Y. nebulosus*, *Y. olmecae*; directed dorsally and anteriorly in *Y. jimenezi*, *Y. mazatecus*; directed anteriorly in *Y. veracrucensis*, *Y. laticornis*; elevated in the posterior half bending abruptly forward in the anterior half in *Y. cameliae*), central tooth not reaching the anterior border of the frontoclypeal suture (shared with *Y. jimenezi*, *Y. imbellis*, *Y. mazatecus*, *Y. nebulosus*, *Y. olmecae*; reaching the clypeus in *Y. laticornis*, *Y. cameliae*, *Y. veracrucensis*); and large eyes (shared with *Y. imbellis*; eyes moderately reduced in *Y. veracrucensis*, *Y. cameliae*, *Y. jimenezi*, *Y. nebulosus*, *Y. olmecae*; strongly reduced in *Y. laticornis*, *Y. mazatecus*).

##### Dimensions

**(mm) (n = 12).** Total length 18.0–21.0 (*χ* = 19.0); elytral length 10.0–11.5 (χ = 11.0); pronotal length 3.5–5.0 (χ = 4.5); pronotal width 5.5–6.0 (χ = 5.5); humeral width 5.5–6.5 (χ = 6.0).

##### Redescription of lectotype

**Figure 5.**
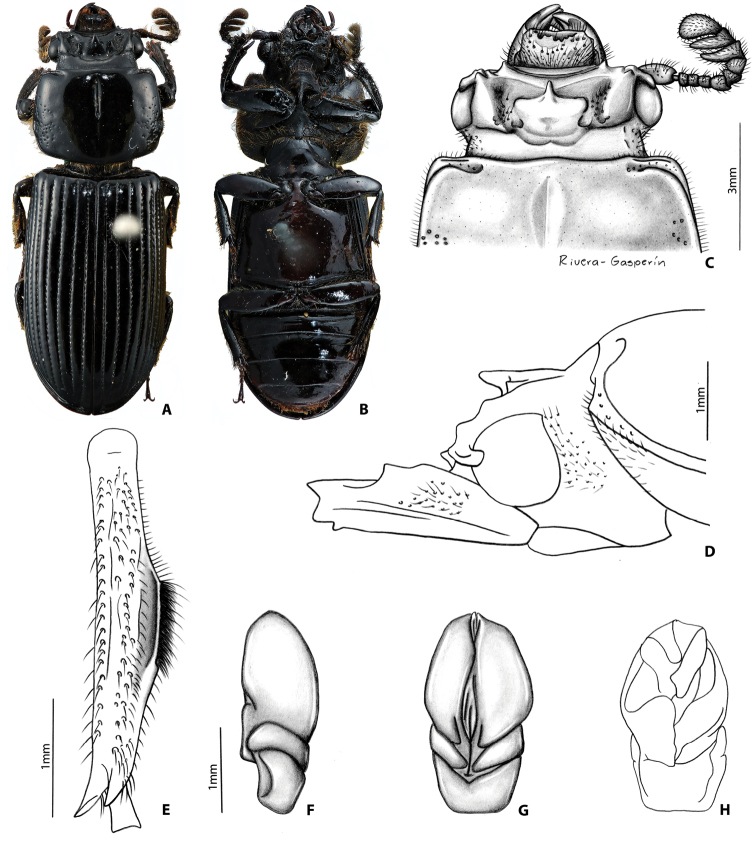
*Yumtaax recticornis* (Burmeister), lectotype: **A** dorsal habitus **B** ventral habitus **C** dorsal view of pronotum and head **D** lateral view of pronotum and head **E** lateral view of right mesotibia **F** lateral view of aedeagus **G** ventral view of aedeagus **H** dorsal view of aedeagus.

**(Fig. 5).** Head (Fig. 5C). Labrum: anterior border concave, dorsal surface smooth and glabrous medially, punctate and setose apicolaterally, apically, and basally; anterior edge excavated. Clypeus: inclined, rectangular, concave, shiny, and smooth. Frontoclypeal suture: concave and opaque; external tubercles rounded, directed anteriorly and laterally. Frontal area: inclined, concave, smooth and shiny; frontal ridges present without inner tubercles. Frontal fossae: punctate and setose. Mesofrontal structure (MFS): of the “falsus” type (see Reyes-Castillo 1970); base subparallel and as wide as the lateral ridge of MFS; center horn short with apex rounded, largely free and directed anteriorly and dorsally (Fig. 5D), not reaching the posterior margin of clypeus, dorsally without micro-punctures; base of center horn wide, narrowing gradually until apex; dorsal fossa present at the base of MFS. Occipital fossa: shallow posteriorly and deeper laterally, connected with the frontal fossae. Posterior occipital sulcus biconcave. Supraorbital ridge: bituberculate, anterior tubercle larger than posterior tubercle; posterior half of supraorbital ridge not bifurcated. Canthus: with apex rounded, covering less than 1/3 of eye, not expanded distally. Eyes large (distal edge of the eye surpassing distal edge of the canthus), width = 0.5 mm (each eye). Head width (between posterior tubercles of the supraorbital ridge) = 3.0 mm. Ratio of sums of both eye widths/total head width = 0.36; postocular area punctate and setose. Ligula: tridentate, central tooth surpassing apex of lateral teeth, lateral teeth rounded; setose punctures present in discal area; posterior border convex. Mentum: lateral lobes rounded and wide, with setose punctures. Basomedial portion protruding ventrally; anterior border at middle convex; basal fossae present and rugose. Hypostomal process: with lateral depression; separated from mentum by a distance shorter than the width of the anterior width of hypostomal process. Infraocular ridge (e.g., Fig. 3): short and narrow anteriorly. Mandible: with 3 apical teeth; internal tooth of left mandible bidentate (teeth 1 and 2 of internal tooth fused); dorsal tooth occupies less than half length of the mandible. Pronotum: anterior angles rounded. Anterior fossae of marginal sulcus punctate. Lateral fossae with scattered punctures. Marginal groove lacking punctures. Prosternum: opaque; prosternellum with anterior half and lateral edges opaque and posterior half and middle area shiny. Scutellum: smooth and glabrous. Mesosternum: with lateral areas opaque. Metasternum: with setae anterolaterally, lacking punctures in lateral margins of metasternal disc. Lateral fossae wide across metathorax, with setose punctures. Elytra: anterior border straight. Meeting point of striae 1-10 (see Reyes-Castillo 1970) with one line of punctures. Wings: well developed. Legs (Fig. 5E): femur I with longitudinal anteroventral groove weakly developed, not reaching distal end of femur, posteroventral half pubescent; setae long, dense, reddish. Abdomen: last sternite with marginal groove complete (Fig. 5B). Aedeagus (Fig. 5F, G, and H) (description based on non-type material): dorsal view phallus elongated (longer than wider). In ventral view distal edges of the phallus more or less at the same level of distal edges of parameres.

##### Variation.

The paralectotype and other specimens vary from the lectotype by the following characteristics: frontoclypeal suture varies from opaque to shiny, from concave to almost straight; frons and clypeus inclined to nearly vertical (always concave); concavity of frons and clypeus vary from strongly developed to nearly flat. Internal tubercles strongly developed or absent; frontal ridges always present, but not always terminating in internal tubercles; ratio of eyes and head width varies from 0.32–0.57; supraocular ridge weakly developed or absent. Occipital sulcus biconcave to concave in a few individuals. Small portion of individuals with frontal ridges fused to the base of the central horn of MFS (apex of the central horn always free).

##### Distribution.

The lectotype and paralectotype are labeled “Mexico” (Burmeister 1847). Castillo and Reyes-Castillo (1984) suggested the Sierra de Juarez in Oaxaca, Mexico, as the possible type locality. The species is known only from cloud forest (1424-2986 m elevation) in Oaxaca, Mexico.

##### Remarks.

Originally, this species was thought to be widely distributed across the Sierra Madre Oriental, the Mexican Transvolcanic Belt, and Sierra Madre del Sur (Reyes-Castillo 1970, Castillo and Reyes-Castillo 1984, Boucher 2006). Our phylogenetic analysis (Fig. 4), as well as close examination of morphological characters, provide evidence that *Y. recticornis* *s. l.* is composed of at least two cryptic species: *Y. recticornis* (“*Y. recticornis* OM” in the *Y. imbellis* clade, Fig. 4 and Suppl. materials 2–4) and *Y. jimenezi* sp. n. (“*Y. recticornis* VM” in the *Y. laticornis* clade, Fig. 4 and Suppl. materials 2–4). Based on comparison with the lectotype and one paralectotype of *Y. recticornis*, this name should be applied to *Y. recticornis* OM. The following character states provide evidence that *Y. recticornis* OM is conspecific with Burmeister’s concept of *Y. recticornis*: large eye size (distal edge of the eye surpassing distal edge of the canthus), shape of the central tooth of the MFS (base subparallel and as wide as the lateral ridge of the MFS; center horn short with apex rounded, largely free and directed anteriorly and dorsally [Fig. 5D]), and shape of the frons and clypeus concave (rather than straight as in *Y. jimenezi*). Castillo and Reyes-Castillo (1984) suggested that eye size variation among *Y. recticornis* *s. l.* was dependent upon the locality of the population (populations in Oaxaca possess large eyes; populations in Veracruz possess small eyes). Characters of the internal tooth of the left mandible and aedeagus are described based on the paralectotype and non-type material because the mandibles of the lectotype are closed (thus making it impossible to determine the state of this character in this specimen).

#### Yumtaax veracrucensis

Taxon classificationAnimaliaColeopteraPassalidae

Beza-Beza, Reyes-Castillo & Jameson sp. n.

http://zoobank.org/E98FFCB6-66DD-4281-9DEF-400E0063359B

##### Material examined.

Seven type specimens (two males, four females, and two sex unknown).

*Holotype* ♂. MEXICO: Veracruz, Municipio de Coatepec, Reserva de La Cortadura, 1895–1900 msnm, bosque mesófilo de montaña, colecta en un tronco podrido, interior del bosque, V-2-2005 (*P. Reyes-Castillo*) (IEXA).

*Paratypes.* MEXICO: 1 ♂, 3 ♀. Veracruz, Municipio de Coatepec, Reserva de La Cortadura, 1895-1900 msnm, bosque mesófilo de montaña, colecta en un tronco podrido, interior del bosque, V-2-2005 (*P. Reyes-Castillo*) (IEXA, CFBB). Chiconquiaco: 1 ♂. Veracruz. Congr. La Guacamaya, X-6-2008 (*P. Rojas*); 2 sex unknown Near La Parra, IX-17-1995 (*J. Bueno*); One paratype is molecular voucher CB0035 (CFBB).

##### Diagnosis.

*Yumtaax veracrucensis* is a small (17.5–20.0 mm), macropterous species that is a member of the *Y. imbellis* clade (Fig. 4). This species is diagnosed by the following character combination: the clypeus is vertical (shared with *Y. laticornis*, *Y. cameliae*; inclined in other members of *Yumtaax*) and with the anterior border straight (shared with other members of *Yumtaax* except for *Y. recticornis* and *Y. olmecae* that have a concave anterior border); mesofrontal structure (MFS) of the “falsus” type (see Reyes-Castillo 1970) (shared with all members of *Yumtaax* except *Y. cameliae* which has the MFS of the “striatopunctatus” type) with the central tooth largely free (shared with *Y. recticornis*, *Y. laticornis*, *Y. cameliae*, *Y. mazatecus*; fused with frontal ridges in *Y. jimenezi*, *Y. imbellis*, *Y. nebulosus*, *Y. olmecae*), directed anteriorly (shared with *Y. laticornis*; directed dorsally in *Y. recticornis*, *Y. imbellis*, *Y. nebulosus*, *Y. olmecae*; directed dorsally and anteriorly in *Y. jimenezi*, *Y. mazatecus*; elevated in the posterior half bending abruptly forward in the anterior half in *Y. cameliae*), and reaching the frontoclypeal suture (shared with *Y. laticornis*, *Y. cameliae*; not reaching the clypeus in other members of *Yumtaax*); and reduced eyes (shared with *Y. cameliae*, *Y. jimenezi*, *Y. nebulosus*, *Y. olmecae*; large in *Y. recticornis*, *Y. imbellis*; strongly reduced in *Y. laticornis*, *Y. mazatecus*).

##### Dimensions

**(mm) (n = 4).** Total length 17.5–20.0 (*χ* = 19.0); elytral length 10.5–11.5 (χ = 11.0); pronotal length 4.0–5.0 (χ = 4.5); pronotal width 5.0–6.5 (χ = 6.0); humeral width 5.0–6.5 (χ = 6.0).

##### Description of holotype

**Figure 6.**
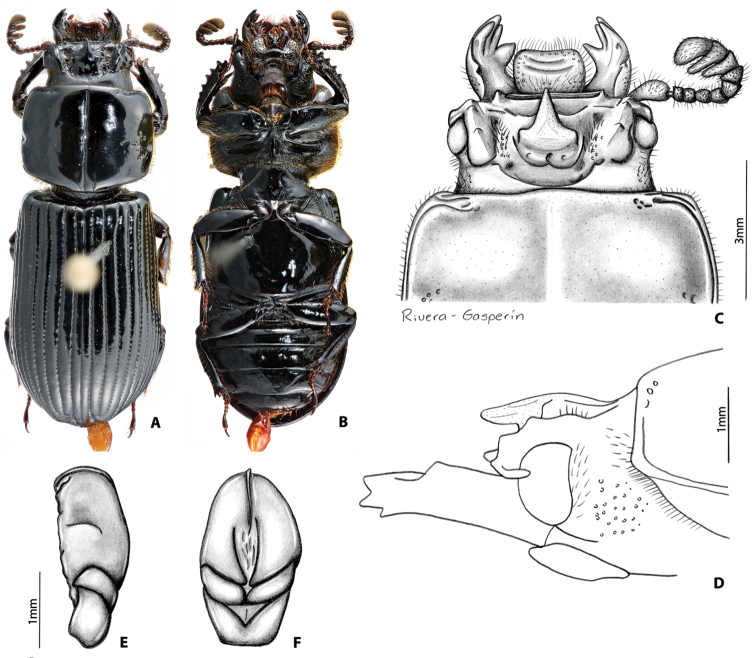
*Yumtaax veracrucensis* Beza-Beza, Reyes-Castillo & Jameson, sp. n., holotype: **A** dorsal habitus **B** ventral habitus **C** dorsal view of pronotum and head **D** lateral view of pronotum and head **E** lateral view of aedeagus **F** ventral view of aedeagus.

**(Fig. 6).** Head (Fig. 6C). Labrum: anterior border concave, dorsal surface smooth and glabrous medially, punctate and setose apicolaterally, apically, and basally; anterior edge excavated. Clypeus: vertical, rectangular, flat, shiny, and smooth. Frontoclypeal suture: straight, and opaque; external tubercles rounded, directed anteriorly and laterally. Frontal area: horizontal, flat, smooth and shiny, frontal ridges weak finishing in inner tubercles; inner tubercles smaller than external tubercles. Frontal fossae: punctate and setose. Mesofrontal structure (MFS): of the “falsus” type (see Reyes-Castillo 1970); base subparallel, slightly narrower than MFS’ lateral ridges; center horn long with apex acute, largely free and directed anteriorly (Fig. 6D), surpassing posterior margin of clypeus, dorsally with sparse micro-punctures; base of the center horn wide, narrowing gradually until apex; dorsal fossa present at the base of MFS. Occipital fossa: shallow posteriorly and deeper laterally connected with the frontal fossae. Posterior occipital sulcus sinuate. Supraorbital ridge: bituberculate, tubercles of similar size; posterior half of supraorbital ridge not bifurcated. Canthus: with apex rounded, almost oblique, covering 1/3 of the eye, not expanded distally. Eyes: reduced (distal edge of the eye not reaching the distal edge of the canthus), width = 0.3 mm (each eye). Head width = 3.0 mm. Ratio of sums of both eyes widths/total head width = 0.2; postocular area punctate and setose. Ligula: tridentate, central tooth surpassing apex of lateral teeth; lateral teeth rounded; setose punctures present in discal area; posterior border convex. Mentum: lateral lobes rounded and wide, with setose punctures. Basomedial portion protruding ventrally; anterior border at the middle convex; basal fossae present with setose punctures. Hypostomal process: without lateral depression; separated from the mentum by a distance shorter than the width of the anterior width of the hypostomal process. Infraocular ridge (e.g., Fig. 3): short, weak, and wide anteriorly. Mandible: with 3 apical teeth; internal tooth in left mandible tridentate; dorsal tooth occupies at least half length of the mandible. Pronotum: anterior angles rounded. Anterior fossae of marginal sulcus punctate. Lateral fossae without punctures. Marginal groove lacking punctures. Prosternum: opaque; prosternellum with anterior and lateral edges rugose and opaque, anteriorly and posteriorly shiny. Scutellum: smooth and glabrous. Mesosternum: with anterior-lateral areas opaque. Metasternum: with setae anterolaterally, lacking punctures in lateral margins of metasternal disc. Lateral fossae wide posteriorly with setose punctuations. Elytra: anterior border straight. Meeting point of striae 1-10 (see Reyes-Castillo 1970) with one line of punctures. Wings: well developed. Legs: femur I with longitudinal anteroventral groove weakly developed, not reaching the distal part of the femur, posteroventral half pubescent; setae long, sparse, reddish. Abdomen: last sternite with marginal groove incomplete (Fig. 6B). Aedeagus (Fig. 6E, F): in dorsal view phallus elongated (longer than wider). In ventral view distal edges of the phallus more or less at the same level of distal edges of parameres.

##### Variation.

Paratypes vary from the holotype by the following characteristics: internal tubercles weak to obsolete; frontal fossae glabrous or setose; ratio of eyes to head width vary between 0.19 and 0.22; basal fossae of mentum strong, opaque and glabrous or shiny and with setose punctures; infraocular ridge weak or absent; femur I with longitudinal antero-ventral groove weakly developed to obsolete.

##### Etymology.

This species is named after its home state of Veracruz in Mexico.

##### Distribution.

This species is known from cloud forest between around 1900 m in the transverse neo-volcanic system, Mexico. The surrounding states and areas in which this species is distributed have been well-collected, and *Y. veracrucensis* has only been found at three localities in Veracruz, Mexico: La Cortadura Natural Reserve near Coatepec; Chiconquiaco (near La Parra); and the road between Las Minas and Xalapa; Chiconquiaco; Congr. La Guacamaya (19°45'51.4"N, 96°48'1.7"W).

##### Remarks.

Specimens of *Y. veracrucensis* were originally identified as *P. orizabae* and were collected in Reserva La Cortadura in Coatepec, Veracruz, Mexico. Based on our phylogenetic analysis, *Y. veracrucensis* (*Y.* LCM) and *Y. imbellis* are potential sister species (Fig. 4; PP 0.99/BS 90). Molecular distinctiveness and form of the dorsal ridge in tibia II (as in all species of *Yumtaax*) provide support that this is a distinct species within the genus *Yumtaax*.

#### Yumtaax laticornis

Taxon classificationAnimaliaColeopteraPassalidae

(Truqui, 1857)

Passalus laticornis Truqui, 1857: 262, 316.
Pseudacanthus laticornis (Truqui) [comb. n. by Kaup 1871: 72, 74].
Petrejoides laticornis (Truqui) [comb. n. by Reyes-Castillo 1970: 125].
Yumtaax laticornis (Truqui) [comb. n. by Boucher 2006: 348].

##### Material examined.

Holotype and 31 non-type specimens.

*Holotype* ♂. MEXICO: Jacale, 1708 (*Sallé*) (BMNH).

*Non-type specimens* (31 total): 2 ♂, 20 ♀, 9 unknown. MEXICO: Veracruz, Calcahualco, Tecuanapa, bosque mesófilo, alt. 2200 m, VI 1992 (*Capistrán, Delgado*) (IEXA; CFBB).

##### Diagnosis.

*Yumtaax laticornis* is a large (24.5–33.0 mm) brachypterous species and is part of the *Yumtaax laticornis* clade (Fig. 4). This species is diagnosed by the following character combination: the clypeus is vertical (shared with *Y. veracrucensis*, *Y. cameliae*; inclined in other members of *Yumtaax*) and the anterior border is straight (shared with other members of *Yumtaax* except for *Y. recticornis* and *Y. olmecae* with concave anterior border of clypeus); mesofrontal structure (MFS) of the “falsus” type (see Reyes-Castillo 1970) (shared with all members of *Yumtaax* except *Y. cameliae* which has the MFS of the “striatopunctatus” type), with the central tooth largely free (shared with *Y. recticornis*, *Y. veracrucensis*, *Y. cameliae*, *Y. mazatecus*; fused with frontal ridges in *Y. jimenezi*, *Y. imbellis*, *Y. nebulosus*, *Y. olmecae*), directed anteriorly (shared with *Y. veracrucensis*; directed dorsally in *Y. recticornis*, *Y. imbellis*, *Y. nebulosus*, *Y. olmecae*; directed dorsally and anteriorly in *Y. jimenezi*, *Y. mazatecus*; elevated in the posterior half bending abruptly forward in the anterior half in *Y. cameliae*), and reaching the frontoclypeal suture (shared with *Y. veracrucensis*, *Y. cameliae*; not reaching the clypeus in other members of *Yumtaax*); eyes are strongly reduced (shared with *Y. mazatecus*; eyes large in *Y. recticornis*, *Y. imbellis*; eyes moderately reduced in *Y. veracrucensis*, *Y. cameliae*, *Y. jimenezi*, *Y. nebulosus*, *Y. olmecae*); and the scutellum is punctate (smooth in other members of *Yumtaax*).

##### Dimensions

**(mm) (n = 19).** Total length 24.5–33.0 (*χ* = 29.5); elytral length 14.0–17.5 (χ = 16.5); pronotal length 6.0–9.0 (χ = 8.0); pronotal width 8.0–11.0 (χ = 10.0); humeral width 7.0–10.0 (χ = 9.0).

##### Redescription of holotype

**Figure 7.**
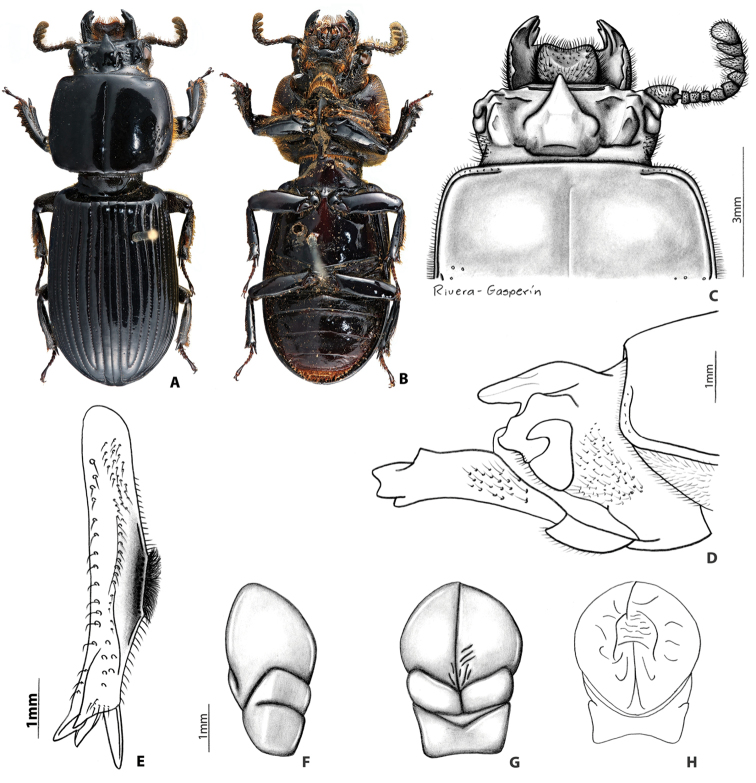
*Yumtaax laticornis* (Truqui), holotype: **A** dorsal habitus **B** ventral habitus **C** dorsal view of pronotum and head **D** lateral view of pronotum and head **E** lateral view of right mesotibia **F** lateral view of aedeagus **G** ventral view of aedeagus **H** dorsal view of aedeagus.

**(Fig. 7).** Head (Fig. 7C). Labrum: anterior border concave, dorsal surface smooth and glabrous medially, punctate and setose apicolaterally, apically, and basally; anterior edge excavated. Clypeus: vertical, rectangular, flat, shiny, and smooth. Frontoclypeal suture: straight, and shiny; external tubercles rounded weak, directed anteriorly. Frontal area: vertical, flat, smooth and shiny, frontal ridge absent. Frontal fossae: punctate and glabrous. Mesofrontal structure (MFS): of the “falsus” type (see Reyes-Castillo 1970); base subparallel and narrower than MFS’ lateral ridge; center horn long with apex acute, largely free and directed anteriorly (Fig. 7D), surpassing posterior margin of clypeus, dorsally without micro-punctures; base of center horn wide narrowing gradually until apex; dorsal fossa absent at the base of MFS. Occipital fossa: shallow posteriorly and deeper laterally, not connected with the frontal fossae. Posterior occipital sulcus concave. Supraorbital ridge: bituberculate, tubercles of similar size; posterior half of supraorbital ridge bifurcated. Canthus: with apex rounded, covering more than 1/3 of the eye, expanded distally. Eyes: strongly reduced (distal edge of the eye shorter than the distal edge of the canthus), width = 0.4 mm (each eye). Head width (between posterior tubercles of the supraorbital ridge) = 5.0 mm. Ratio of sums of both eyes widths/total head width = 0.16; postocular area punctate and setose. Ligula: tridentate, central tooth surpassing apex of lateral teeth, lateral teeth rounded; setose punctures present in discal area; posterior border straight. Mentum: lateral lobes rounded and wide, with setose punctures. Basomedial portion protruding ventrally; anterior border at middle straight; basal fossae present, with setose punctures. Hypostomal process: with lateral depression; separated from mentum by a distance larger than the wide of the anterior width of hypostomal process. Infraocular ridge (e.g., Fig. 3): short and wide anteriorly, narrow posteriorly. Mandible: with 3 apical teeth; internal tooth of left mandible bidentate; dorsal tooth occupies more than half length of the mandible. Pronotum: anterior angles rounded. Anterior fossae of marginal sulcus punctate. Lateral fossae without punctures. Marginal groove with punctures. Prosternum: opaque. Prosternellum with anterior half and lateral edges opaque and posterior half and middle area shiny. Scutellum: punctate and glabrous. Mesosternum: with anterior-lateral areas opaque. Metasternum: with setae anterolaterally, lacking punctures in lateral margins of the metasternal disc. Lateral fossae wide glabrous posteriorly with setose punctures anteriorly. Elytra: anterior border straight. Meeting point of striae 1-10 (see Reyes-Castillo 1970) with one line of punctures. Wings: reduced. Legs (Fig. 7E): femur I with longitudinal anteroventral groove strongly developed, reaching the distal end of the femur, posterioventral half pubescent; setae short, dense, reddish. Abdomen: last sternite with marginal groove complete (Fig. 7B). Aedeagus (Fig. 7F, G, and H) (Description based on non-type material): dorsal view phallus globose (wider than long). Ventral view lateral edges of the phallus surpassing the laterodistal edges of the parameres.

##### Variation.

The non-type material differs from the holotype in the following characters: internal tubercles obsolete to strongly developed; frontal ridges obsolete to strongly developed; frontal area glabrous to sparsely setose; ratio of eyes versus head width varies from 0.13-0.23; pronotum laterally with or without strong punctures, even at the individual level (right vs left side of the pronotum); prosternelum completely opaque or opaque and shiny.

##### Distribution.

This species is known from cloud forest (bosque mesófilo, 2200 m elevation) at Orizaba Peak, Veracruz, Mexico. In the original description, Truqui (1857) cited one specimen collected by Sallé from Jacal near the Orizaba Volcano. This locality corresponds to El Jacal, Coscomatepec, Orizaba Peak (Reyes-Castillo 2011).

MEXICO: Veracruz: Calcahualco (Tecuanapa, road from Calcahualco to the Pico de Orizaba), Jacale, Pico de Orizaba.

##### Remarks.

Castillo and Reyes-Castillo (1984) redescribed *Y. laticornis* without examining type specimens. We compared two specimens of *Y. laticornis* determined by Castillo and Reyes-Castillo with the holotype specimen designated by M. E. Bachus at The Natural History Museum, London. Close examination of the holotype and results of the phylogenetic analysis (Fig. 4 and Suppl. materials 2–4) provide evidence that *Y. laticornis* is not conspecific with *Y. laticornis* sensu Castillo and Reyes-Castillo (1984) (*Y.* LM in Suppl. materials 2–4). Specimens described as *Y. laticornis* by Castillo and Reyes-Castillo (1984) correspond with *Y. cameliae* sp. n. (*Y.* LM in Suppl. materials 2–4), and the holotype of *Y. laticornis* corresponds with *Y.* CM (Suppl. materials 2–4). The overall length of the holotype specimen is 30.0 mm, and this falls within the size range for *Y. laticornis* (25.0–33.0 mm), but not within the range for *Y. cameliae* (22.5–25.5 mm). Furthermore, based on distribution and biogeography, *Y. cameliae* has been collected only in the type locality where suitable habitat for the species occurs. This area is geographically isolated from the distribution area of *Y. laticornis*. *Yumtaax laticornis*’ inclusion in the *Y. laticornis* clade (is strongly supported (PP 1.0/BS 100) (Fig. 4).

#### Yumtaax cameliae

Taxon classificationAnimaliaColeopteraPassalidae

Beza-Beza, Reyes-Castillo & Jameson sp. n.

http://zoobank.org/FAEEDD32-CA2C-4FD5-99D8-3A4A73DDC04F

##### Material examined.

22 type specimens.

*Holotype* ♀. MEXICO: Veracruz, Acultzingo, Puerto del Aire, 2400 msnm, bosque mesófilo de montaña, encinar tronco 4, VII-16-80 (*C. Castillo*) (IEXA).

*Paratypes* (21 total). 1 ♂, 7 ♀ with same label data as holotype. MEXICO: 3 ♀, Veracruz, Acultzingo, VI-I-1963 (*G. Halffter*) (IEXA). 1 ♀, Acultzingo, Puerto del Aire, 2400 msnm, bosque mesófilo de montaña, encinar tronco 4, VII-17-80 (*C. Castillo*) (IEXA, CFBB). 2 ♂, 3 ♀, Acultzingo, Puerto del Aire, 2400 msnm, bosque mesófilo de montaña, encinar tronco 4, VIII-16-80 (*C. Castillo*) (IEXA, CFBB). 4 ♀, Acultzingo, Puerto del Aire, 2400 msnm, bosque mesófilo de montaña, encinar tronco 4, VIII-17-80 (*C. Castillo*) (IEXA, CFBB).

##### Diagnosis.

*Yumtaax cameliae* is a medium sized (22.5–25.5 mm), macropterous species that is part of the *Y. laticornis* clade (Fig. 4). This species is diagnosed by the following character combination: the clypeus is vertical (shared with *Y. laticornis*, *Y. veracrucensis*; inclined in other members of *Yumtaax*) and with the anterior border straight (shared with other members of *Yumtaax* except for *Y. recticornis* and *Y. olmecae* with concave anterior border of clypeus); mesofrontal structure (MFS) of the “striatopunctatus” type (see Reyes-Castillo 1970) (MFS of the “falsus” type in other members of *Yumtaax*), with the central tooth largely free (shared with *Y. recticornis*, *Y. laticornis*, *Y. veracrucensis*, *Y. mazatecus*; fused with frontal ridges in *Y. jimenezi*, *Y. imbellis*, *Y. nebulosus*, *Y. olmecae*), elevated in the posterior half and bending abruptly forward in the anterior half (directed dorsally *Y. recticornis*, *Y. imbellis*, *Y. nebulosus*, *Y. olmecae*; directed dorsally and anteriorly in *Y. jimenezi*, *Y. mazatecus*; directed anteriorly in *Y. veracrucensis*, *Y. laticornis*), reaching the clypeus (shared with *Y. veracrucensis*, *Y. cameliae*; not reaching the clypeus in other members of *Yumtaax*); and moderately reduced eyes (shared with *Y. veracrucensis*, *Y. jimenezi*, *Y. nebulosus*, *Y. olmecae*; large in *Y. recticornis*, *Y. imbellis*; strongly reduced in *Y. laticornis*, *Y. mazatecus*).

##### Dimensions

**(mm) (n = 4).** Total length 22.5–25.5 (*χ* = 24.0); elytral length 13.5–14.0 (χ = 14.5); pronotal length 6.0–7.0 (χ = 6.5); pronotal width 7.0–9.5 (χ = 8.5); humeral width 7.0–8.0 (χ = 7.5).

##### Description of holotype

**Figure 8.**
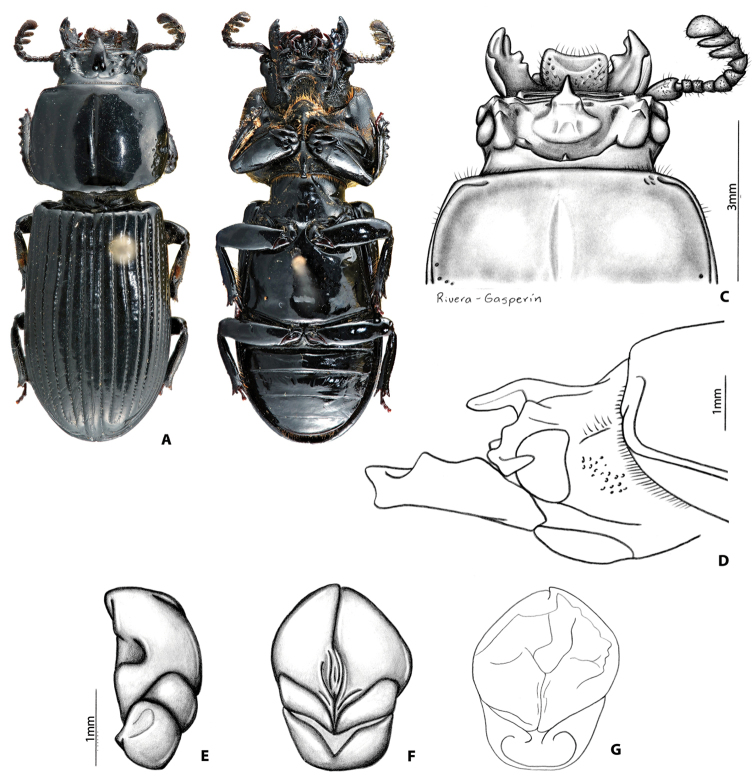
*Yumtaax cameliae* Beza-Beza, Reyes-Castillo & Jameson, sp. n., holotype: **A** dorsal habitus **B** ventral habitus **C** dorsal view of pronotum and head **D** lateral view of pronotum and head **E** lateral view of aedeagus **F** ventral view of aedeagus **G** dorsal view of aedeagus.

**(Fig. 8).** Head (Fig. 8C). Labrum: anterior border concave, dorsal surface smooth and glabrous medially, punctate and setose apicolaterally, apically, and, basally; anterior edge excavated. Clypeus: vertical, rectangular, flat, shiny, and smooth. Frontoclypeal suture: straight, and shiny; external tubercles rounded, weak, directed dorsally and anteriorly. Frontal area: inclined, flat, smooth, and shiny; frontal ridges absent without inner tubercles. Frontal fossae: impunctate and glabrous. Mesofrontal structure (MFS): of the “striatopunctatus” type (see Reyes-Castillo 1970); base subparallel and narrower than MFS’ lateral ridge; center horn long with apex acute, largely free elevated in the posterior half bending abruptly forward in the anterior half (Fig. 8D), reaching the posterior margin of clypeus, dorsally without micro-punctures; base of center horn wide, not narrowing in the posterior half and narrowing abruptly in the anterior half until apex; dorsal fossa present at base of MFS. Occipital fossa: shallow posteriorly and deeper laterally connected with the frontal fossae. Posterior occipital sulcus concave. Supraorbital ridge: bituberculate, tubercles of similar size; posterior half of supraorbital ridge not bifurcated. Canthus: with apex rounded, covering less than 1/3 of the eye, not expanded distally. Eyes moderately reduced (distal edge of the eye more or less at the distal edge of the canthus), width = 0.6 mm (each eye). Head width (between posterior tubercles of the supraorbital ridge) = 4.3 mm. Ratio of sums of both eyes widths/total head width = 0.27; postocular area punctate and setose. Ligula: tridentate, central tooth surpassing apex of lateral teeth; lateral teeth rounded; glabrous punctures present in discal area; posterior border straight. Mentum: lateral lobes rectangular and wide, with setose punctures. Basomedial portion protruding ventrally; anterior border at middle convex; basal fossae present. Hypostomal process: without lateral depression; separated from mentum by a distance shorter than the width of the anterior width of hypostomal process. Infraocular ridge (Fig. 3): short and wide anteriorly, narrow posteriorly. Mandible: with 3 apical teeth; internal tooth of left mandible bidentate; dorsal tooth occupies half of length of the mandible. Pronotum: anterior angles rounded. Anterior fossae of marginal sulcus punctate. Lateral fossae impunctate. Marginal groove lacking punctures. Prosternum: opaque; prosternellum with anterior half and lateral edges opaque and posterior half and middle area shiny. Scutellum: smooth and glabrous. Mesosternum: with anterolateral areas opaque. Metasternum: with setae anterolaterally, lacking punctures in lateral margins of metasternal disc. Lateral fossae wide posteriorly with setose punctures. Elytra: anterior border straight. Meeting point of striae 1-10 (see Reyes-Castillo 1970) with one line of punctures. Wings: well developed. Legs: femur I with longitudinal anteroventral groove strongly developed, reaching distal end of femur, posteroventral half pubescent; setae long, sparse, reddish. Abdomen: last sternite with marginal groove complete and opaque laterally (Fig. 8B). Aedeagus (Fig. 8E, F, G) (based on male paratype): in dorsal view phallus globose (wider than long). In ventral view distal edges of phallus surpassing the distal edge of the parameres.

##### Variation.

Paratypes vary from the holotype in the following characters: internal tubercles obsolete to strongly developed; frontal ridges obsolete to strongly developed; frontal area glabrous to setose; ratio of eyes versus head width varies from 0.19-0.31; central area of the ligula always punctate, occasionally setose; pronotum with lateral fossae with or without strong punctures, even at the individual level (right vs left side of the pronotum); prosternellum shiny (one specimen of the type series) or opaque in anterior half; terminal sternite with lateral areas of the marginal groove opaque or not.

##### Etymology.

The species is named *Y. cameliae*, honoring Passalidae researcher Camelia Castillo whose research (Castillo and Reyes-Castillo 1984) provided a better understanding of *Yumtaax*.

##### Distribution.

This species is known only from the type locality in Veracruz, Mexico. It was collected in a small patch of oak forest (bosque mesófilo de montaña) surrounding the Puerto del Aire village at 2400 m elevation.

##### Remarks.

Specimens of *Y. cameliae* were originally identified as *Y. laticornis* (9A in Castillo and Reyes-Castillo 1984). Close examination of the *Y. laticornis* holotype (see “Remarks” for *Y. laticornis*) and distribution of the holotype suggested that *Y. laticornis* sensu Castillo and Reyes-Castillo and *Y. laticornis* Truqui do not correspond to the same species.

#### Yumtaax jimenezi

Taxon classificationAnimaliaColeopteraPassalidae

Beza-Beza, Reyes-Castillo & Jameson sp. n.

http://zoobank.org/C8313A69-5326-49BF-829B-53343207F53E

##### Material examined.

27 type specimens.

*Holotype* ♂. MEXICO: Veracruz, Calcahualco, Tecuanapa. Bosque mesófilo, alt. 2400 m V-2/3-1992 (*Capistrán and Delgado*) (IEXA).

*Paratypes* (26 total). MEXICO: Veracruz: 10 ♀, 15 unknown sex, Calcahualco, Tecuanapa, bosque mesófilo, alt. 2400 m, V-2/3-1992 (*Capistrán and Delgado*). 5 ♂, 8 ♀, 34 sex unknown, Calcahualco, Tecuanapa, bosque mesófilo, alt. 2400 m, V-1992 (*Capistrán and Delgado*). 14 ♀, Calcahualco, Tecuanapa, bosque mesófilo, alt. 2200 m, VI-1992 (*Capistrán and Delgado*). 1 ♂, Calcahualco, Dos Caminos, II-29-1992, alt. 1415 m, bosque de encino-pino, dentro de *Quercus* sp. (*R. Novelo, F. Capistrán and L. Delgado*). 1 ♀, 2 sex unknown, Calcahualco, Nueva Vaquería (1 km before), II-28-1992, alt. 2700 m, bosque de pino-encino, en tronco (*R. Novelo, F. Capistrán and L. Delgado*). 2 ♀, Veracruz, Calcahualco, 1 km antes de Nueva Vaquería, 2700 m, VI-1992, (*L. Delgado and Capistrán*) (CFBB, IEXA). 1 ♂, Cosautlan, Los Laureles, alt. 2680 m, VIII-27-1999 (*J. P. Lumaret*). 4 sex unknown, Orizaba, Sallé, Mex. Collection (*Sallé*) (BMNH). 1 sex unknown, Mexico (*Truqui*) (BMNH).

##### Diagnosis.

*Yumtaax jimenezi* is a small (18.5–23.0 mm) macropterous species, and it is part of the *Y. laticornis* clade (=Fig. 4). This species is diagnosed by the following character combination: clypeus is inclined (shared with *Y. recticornis*, *Y. imbellis*, *Y. mazatecus*, *Y. nebulosus*, *Y. olmecae*; vertical in *Y. laticornis*, *Y. cameliae*, *Y. veracrucensis*) and with the anterior border straight (shared with other members of *Yumtaax* except for *Y. recticornis* and *Y. olmecae* that have the anterior border of clypeus concave); MFS of the “falsus” type (see Reyes-Castillo 1970) (shared with all members of *Yumtaax* except *Y. cameliae* which has the MFS of the “striatopunctatus” type), with the central tooth that is not free (fused with frontal ridges) (shared with *Y. imbellis*, *Y. nebulosus*, *Y. olmecae*; largely free in *Y. recticornis*, *Y. veracrucensis*, *Y. laticornis*, *Y. cameliae*, *Y. mazatecus*), directed dorsally and anteriorly (shared with *Y. mazatecus*, directed dorsally *Y. recticornis*, *Y. imbellis*, *Y. nebulosus*, *Y. olmecae*; directed anteriorly in *Y. veracrucensis*, *Y. laticornis*; elevated in the posterior half bending abruptly forward in the anterior half in *Y. cameliae*), and not reaching the clypeus (shared with *Y. recticornis*, *Y. imbellis*, *Y. mazatecus*, *Y. nebulosus*, *Y. olmecae*; reaching the clypeus in *Y. laticornis*, *Y. cameliae*, *Y. veracrucensis*); and moderately reduced eyes (shared with *Y. veracrucensis*, *Y. cameliae*, *Y. nebulosus*, *Y. olmecae*; large in *Y. recticornis*, *Y. imbellis*; strongly reduced in *Y. laticornis*, *Y. mazatecus*).

##### Dimensions

**(mm) (n = 12).** Total length 18.5–23.0, (*χ* = 20.5); elytral length 11.5–14.0, (χ = 12.5); pronotal length 4.0–6.0, (χ = 5.5); pronotal width 5.5–7.0, (χ = 6.5); humeral width 5.5–7.0, (χ = 6.5).

##### Description of holotype

**Figure 9.**
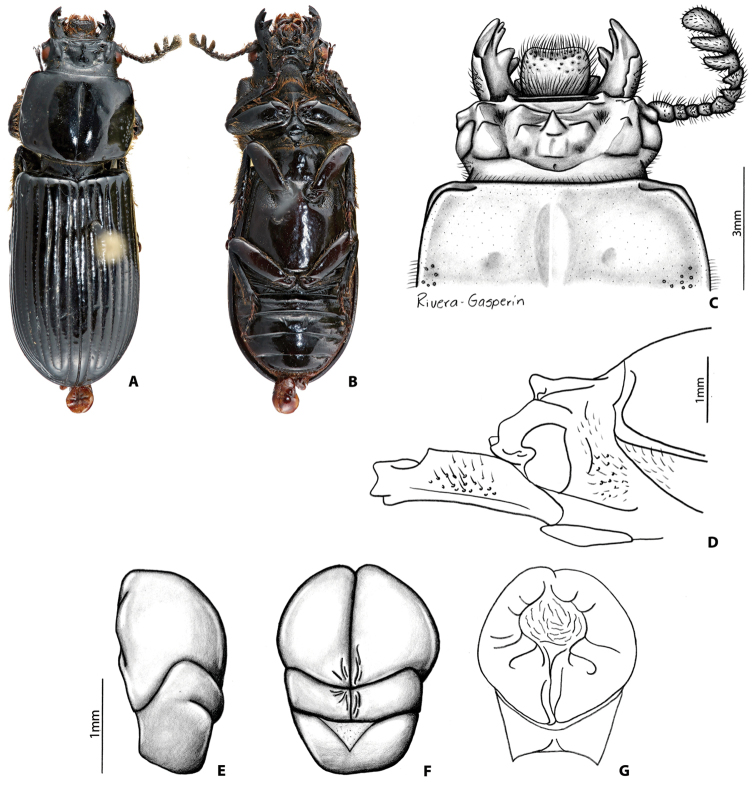
*Yumtaax jimenezi* Beza-Beza, Reyes-Castillo & Jameson, sp. n., holotype: **A** dorsal habitus **B** ventral habitus **C** dorsal view of pronotum and head **D** lateral view of pronotum and head **E** lateral view of aedeagus **F** ventral view of aedeagus **G** dorsal view of aedeagus.

**(Fig. 9).** Head (Fig. 9C). Labrum: anterior border concave, dorsal surface smooth and glabrous medially, and punctate and setose in the apicolaterally, apically, and basally; anterior edge excavated. Clypeus: inclined, rectangular, shiny, and smooth. Frontoclypeal suture: straight, and shiny. External tubercles rounded and directed dorsally. Frontal area: inclined, smooth, and shiny, frontal ridges present finishing in inner tubercles. Frontal fossae: punctate and setose. Mesofrontal structure (MFS): of the “falsus” type (see Reyes-Castillo 1970); base subparallel and narrower than the MFS’ lateral ridge; center horn short with apex rounded, not free (fused with frontal ridges) and directing dorsally (Fig. 9D), not reaching the posterior margin of clypeus (Fig. 9C, D), dorsally without micro-punctures; base of the center horn narrow not narrowing down along its length (central tooth tubercle like shape [Fig. 9C, D]); dorsal fossa present at the base of MFS. Occipital fossa: shallow posteriorly and deeper laterally not connected to frontal fossae. Posterior occipital sulcus sinuate. Supraorbital ridge: bituberculate, tubercles of similar size; posterior half of supraorbital ridge not bifurcated. Canthus: with apex rounded covering less than 1/3 of the eye, expanded distally. Eyes: reduced (distal edge of the eye more or less at the distal edge of the canthus), width = 0.5 mm (each eye). Head width = 3.5 mm. Ratio of sums of both eyes widths/total head width = 0.24; postocular area punctate and setose. Ligula: tridentate, with central tooth surpassing apex of lateral teeth, lateral teeth rounded; setose punctures present in discal area; posterior border convex. Mentum: lateral lobes rounded and wide, with setose punctures. Basomedial portion protruding ventrally; anterior border at middle convex; basal fossae absent. Hypostomal process: without lateral depression; separated from mentum by a distance shorter than the wide of the anterior width of hypostomal process. Infraocular ridge absent. Mandible: with 3 apical teeth; internal tooth of left mandible bidentate; dorsal tooth occupies less than half length of the mandible. Pronotum: anterior angles rounded. Anterior fossae of marginal sulcus impunctate. Lateral fossae with heavy punctures. Marginal groove lacking punctures. Prosternum: opaque. Prosternellum anterior half opaque and lateral edges and posterior half shiny. Scutellum: smooth and glabrous. Mesosternum: with anterolateral areas opaque. Metasternum: with setae in anterolaterally, without punctures in lateral margins of metasternum disc. Lateral fossae wide across the metathorax, with setose punctures. Elytra: anterior border straight. Meeting point of striae 1-10 (see Reyes-Castillo 1970) with one line of punctures. Wings: well developed. Legs: femur I with longitudinal anteroventral groove weakly developed in the proximal half and strongly developed on the distal end of the femur, posteroventral half pubescent; setae long, sparse, reddish. Abdomen: last sternite with marginal groove complete (Fig. 9B). Aedeagus (Fig. 9E, F, G): in dorsal view phallus globose (wider than long). In ventral view distal edges of phallus surpassing the distal edge of the parameres.

##### Variation.

Frontoclypeal suture can be from opaque to shiny; internal tubercles from strongly to weakly marked but always present; ratio of eyes and head with varies from 0.18-0.32; supraocular ridge from weak to absent; hypostomal process with weak lateral depression to lateral depression absent; prosternellum varies from anterior half opaque and lateral edges and posterior half shiny to anterior half and lateral edges opaque and posterior half and middle shiny to completely opaque; femur I longitudinal anterior-ventral groove from weak in the proximal half to absent; femur I longitudinal anterior-ventral groove from strongly developed in the distal half to absent.

##### Etymology.

This species is named in honor of Passalidae worker Dr. Larry Jiménez-Ferbans who assisted in collecting trips supporting this study.

##### Distribution.

This species is known from cloud forest (bosque mesófilo) at 2400 m elevation from the state of Veracruz, Mexico.

MEXICO: Veracruz: Calcahualco (Tecuanapa, Dos Caminos, Nueva Vaquería [1 km before]).

##### Remarks.

*Yumtaax jimenezi* is a cryptic, widespread species that has been confused with *Y. recticornis*. Previously, *Y. recticornis* *s. l.* was thought to be broadly distributed in Mexico from the Sierra Madre Oriental in the Mexican Transvolcanic Belt and Sierra Madre del Sur (Reyes-Castillo 1970, Castillo and Reyes-Castillo 1984, Boucher 2006). Phylogenetic analysis (Fig. 4 and Suppl. materials 2–4) and close examination of morphology provide evidence that *Y. recticornis* *s. l.* comprises two cryptic species [*Y. recticornis* (= *Y. recticornis* OM) and *Y. jimenezi* (*Y. recticornis* VM)].

These species are distinguished by eye size (small in *Y. jimenezi* and large in *Y. recticornis*), shape of the central tooth of the MFS (center horn short with apex rounded, not free [fused with frontal ridges] and directed dorsally [Fig. 9D] in *Y. jimenezi*; center horn short with apex rounded, largely free and directed anteriorly and dorsally [Fig. 5D] in *Y. recticornis*), and the shape of the surface of the frons and clypeus (concave in *Y. recticornis* versus flat in *Y. jimenezi*). Interestingly, the reduced eye size in *Y. jimenezi* results in the distal expansion of the canthus. Based on seven exemplars, phylogenetic analysis (Fig. 4) strongly supports *Y. jimenezi* as a unique lineage (1.0 PP/100 BS).

## Conclusions

A single, unique synapomorphy (dorsal mesotibial ridge elevated at the middle), molecular phylogenetic analysis, and distributional affinities collectively support the hypothesis of *Yumtaax* monophyly.

*Yumtaax* species, as with most Passalidae, exhibit a high degree of morphological conservatism, rendering traditional systematics studies quite challenging. Cryptic species, such as those revealed in this study, are likely to be discovered by employing molecular data and careful consideration of morphological characters. Further studies, ideally those that include significant additional molecular phylogenetic data, are needed to rigorously evaluate the Passalidae species boundaries and evolutionary history.

## Supplementary Material

XML Treatment for Yumtaax recticornis

XML Treatment for Yumtaax veracrucensis

XML Treatment for Yumtaax laticornis

XML Treatment for Yumtaax cameliae

XML Treatment for Yumtaax jimenezi

## Supplementary materials

Supplementary material 1 Table S1 Data type: specimen data Explanation note: Voucher specimens for taxa included in the molecular analysis (species, depository, preservation method, collection data, and GenBank DNA sequence accession numbers). This dataset is made available under the Open Database License (http://opendatacommons.org/licenses/odbl/1.0/). The Open Database License (ODbL) is a license agreement intended to allow users to freely share, modify, and use this Dataset while maintaining this same freedom for others, provided that the original source and author(s) are credited.Cristian Fernando Beza-Beza, James Beck, Pedro Reyes-Castillo, Mary Liz Jameson

Supplementary material 2 Figure S1 Data type: molecular data Explanation note: 50% majority-rule consensus of Bayesian posterior probabilities resulting from analysis of the COI data. Bayesian posterior probabilities (PP) (> 0.50) and bootstrap support (BS) (> 50) are noted. *Yumtaax veracrucensis* sp. n. = *Yumtaax* LCM; *Yumtaax recticornis* = *Yumtaax recticornis* OM; *Yumtaax laticornis* = *Yumtaax* CM; *Yumtaax cameliae* sp. n. = *Yumtaax* LM; *Yumtaax jimenezi* sp. n. = *Yumtaax recticornis* VM.. This dataset is made available under the Open Database License (http://opendatacommons.org/licenses/odbl/1.0/). The Open Database License (ODbL) is a license agreement intended to allow users to freely share, modify, and use this Dataset while maintaining this same freedom for others, provided that the original source and author(s) are credited.Cristian Fernando Beza-Beza, James Beck, Pedro Reyes-Castillo, Mary Liz Jameson

Supplementary material 3 Figure S2 Data type: molecular data Explanation note: 50% majority-rule consensus of Bayesian posterior probabilities resulting from analysis of the 12S data. Bayesian posterior probabilities (PP) (> 0.50) and bootstrap support (BS) (> 50) are noted. *Yumtaax veracrucensis* sp. n. = *Yumtaax* LCM; *Yumtaax recticornis* = *Yumtaax recticornis* OM; *Yumtaax laticornis* = *Yumtaax* CM; *Yumtaax cameliae* sp. n. = *Yumtaax* LM; *Yumtaax jimenezi* sp. n. = *Yumtaax recticornis* VM. This dataset is made available under the Open Database License (http://opendatacommons.org/licenses/odbl/1.0/). The Open Database License (ODbL) is a license agreement intended to allow users to freely share, modify, and use this Dataset while maintaining this same freedom for others, provided that the original source and author(s) are credited.Cristian Fernando Beza-Beza, James Beck, Pedro Reyes-Castillo, Mary Liz Jameson

Supplementary material 4 Figure S3 Data type: molecular data Explanation note: 50% majority-rule consensus of Bayesian posterior probabilities resulting from analysis of the 28S data. Bayesian posterior probabilities (PP) (> 0.50) and bootstrap support (BS) (> 50) are noted. *Yumtaax veracrucensis* sp. n. = *Yumtaax* LCM; *Yumtaax recticornis* = *Yumtaax recticornis* OM; *Yumtaax laticornis* = *Yumtaax* CM; *Yumtaax cameliae* sp. n. = *Yumtaax* LM; *Yumtaax jimenezi* sp. n. = *Yumtaax recticornis* VM. This dataset is made available under the Open Database License (http://opendatacommons.org/licenses/odbl/1.0/). The Open Database License (ODbL) is a license agreement intended to allow users to freely share, modify, and use this Dataset while maintaining this same freedom for others, provided that the original source and author(s) are credited.Cristian Fernando Beza-Beza, James Beck, Pedro Reyes-Castillo, Mary Liz Jameson
