# Supplementary material for: Phylogeny of the genus Yumtaax Boucher (Coleoptera, Passalidae, Proculini): Taxonomic and evolutionary implications with descriptions of three new species
Source: Zookeys. 2017 Apr 10;(667):95–129. doi: 10.3897/zookeys.667.10716 (PMC5523388; doi:10.3897/zookeys.667.10716)
Supplement: Supplementary material 1 — Table S1 [file zookeys-667-095-s001.docx]

**Table S1.** Voucher specimens for taxa included in the molecular analysis (species, depository, preservation method, collection data, and GenBank DNA sequences accession numbers).

| **Voucher** | **Species** | **Depository** | **Preservation** | **Label Data** | **12S** | **28S** | **CO1** |
| --- | --- | --- | --- | --- | --- | --- | --- |
| CB001 | *Yumtaax jimenezi* | WICH | EtOH (-80) | MEXICO: Veracruz. Road El encinal 2 to Ingenio el Rosario Coatepec. N 19º 31’’ 33.4’’ W 97º 4’ 41.4’’. VII-14-2012. | KY671322 | KY671359 | KY671395 |
| CB002 | *Yumtaax jimenezi* | WICH | EtOH (-80) | MEXICO: Veracruz. Road El encinal 2 to Ingenio el Rosario Coatepec. N 19º 31’’ 33.4’’ W 97º 4’ 41.4’’. VII-14-2012. | KY671323 | KY671360 | KY671396 |
| CB003 | *Yumtaax imbelis* | WICH | EtOH (-80) | MEXICO: Guerrero. Tecpan de Galeaya, reserva la mona, Ejido Cordon grande. N 17º 34’ 10.6’’ W 100º 36’ 22.4’’. I-31-2012 | KY671324 | KY671361 | KY671397 |
| CB004 | *Yumtaax mazatecus* | WICH | EtOH (-80) | MEXICO: Oaxaca. Huahuatla de Jimenez. N 18º 10’ 33.9’’ W 97º 0’ 20.8’’. VII-17-2012. | KY671325 | KY671362 | KY671398 |
| CB005 | *Yumtaax jimenezi* | WICH | EtOH (-80) | MEXICO: Veracruz. Municipio Acajete, El encinal 2. N 19º 01’ 04.1’’ W 97º 3’ 6.1’.’ VII-14-2012. | KY671326 | KY671363 | KY671399 |
| CB006 | *Yumtaax jimenezi* | WICH | EtOH (-80) | MEXICO: Veracruz. Municipio Acajete, arriba del Zapotal. N 90º 30’ 48.24’’ W 97º 02’ 25’’. VII-14-2012. | KY671327 | KY671364 | KY671400 |
| CB007 | *Yumtaax jimenezi* | WICH | EtOH (-80) | MEXICO: Veracruz. Calcahualco, Tecuanapa. N 19º 6’ 58.2’’ W 97º 10’ 24.4’’. VII-16-2012. | KY671328 | KY671365 | KY671401 |
| CB008 | *Yumtaax recticornis* | WICH | EtOH (-80) | MEXICO: Oaxaca. Municipio Valle Nacional. N 17º 35’ 25.3’’ W 96º 28’ 45.2’’. VII-18-2012. | KY671329 | KY671366 | KY671402 |
| CB010 | *Petrejoides orizabae* | WICH | EtOH (-80) | MEXICO: Puebla. Cuetzalan del progreso, delante de Iztahuaca. N 20º 1’ 19.6’’ W 97º 30’ 10.6’’. | KY671330 | KY671367 | KY671403 |
| CB011 | *Hesliscus tropicus* | WICH | EtOH (-80) | MEXICO: Veracruz. El mirador. VII-7-2012. | KY671331 | KY671368 | KY671404 |
| CB012 | *Odontotaenius zodiacus* | WICH | EtOH (-80) | MEXICO: Veracruz. Municipio Acajete, arriba d el Zapotal. N 90º 30’ 48.24’’ W 97º 02’ 25’’. VII-14-2012. | KY671332 | KY671369 | KY671405 |
| CB013 | *Verres hageni* | WICH | EtOH (-80) | GUATEMALA: Alta Verapaz. San Juan Chamelco, Chamil. VI-2012. | KY671333 | KY671370 | KY671406 |
| CB014 | *Oileus bifidus* | WICH | EtOH (-80) | MEXICO: Oaxaca. Municipio Valle Nacional. N 17º 36’ 11.9’’ W 96º 22’ 9.4’’. VII-18-2012. | KY671334 | KY671371 | KY671407 |
| CB015 | *Vindex* sp. | WICH | EtOH (-80) | GUATEMALA: Baja Verapaz. Road to Panimaquito, Beza. VI-16-2012. | KY671335 | KY671372 | KY671408 |
| CB016 | *Passalus caelatus* | WICH | EtOH (-80) | GUATEMALA: Baja Verapaz. Purulha, Panimaquito. II-18-2012. |  | KY671373 | KY671409 |
| CB017 | *Yumtaax jimenezi* | WICH | EtOH (-80) | MEXICO: Veracruz. Municipio Acajete, El encinal 2. N 19º 01’ 04.1’’ W 97º 3’ 6.1’’. 2529 msnm. VII-14-2012. Col: Reyes, Beza, and Villerias | KY671336 | KY671374 | KY671410 |
| CB018 | *Yumtaax jimenezi* | WICH | EtOH (-80) | MEXICO: Veracruz. Municipio de Calcahualco, Tecuanapa. N 19º 6’ 58.2’’ W 97º 10’ 24.4’’. 2039 msnm. VII-16-2012. Col: Reyes, Bexa, Villerias, and Jimenez | KY671337 | KY671375 | KY671411 |
| CB019 | *Yumtaax recticornis* | WICH | EtOH (-80) | MEXICO: Oaxaca. Municipio Valle Nacional. N 17º 35’ 25.3’’ W 96º 28’ 45.2’’. 2336 msnm. VII-18-2012. Col: Reyes, Bexa, Villerias, and Jimenez | KY671338 | KY671376 | KY671412 |
| CB020 | *Yumtaax cameliae* | WICH | EtOH (-80) | MEXICO: Veracruz. Acutzingo, Puerto del Aire | KY671339 | KY671377 | KY671413 |
| CB021 | *Yumtaax imbelis* | WICH | EtOH (-80) | MEXICO: Guerrero. | KY671340 | KY671378 | KY671414 |
| CB022 | *Petrejoides guatemalae* | UVG | EtOH (-80) | GUATEMALA: Huehuetenango. | KY671341 | KY671379 | KY671415 |
| CB023 | *Petrejoides guatemalae* | UVG | EtOH (-80) | GUATEMALA: Huehuetenango. | KY671342 | KY671380 | KY671416 |
| CB027 | *Heliscus vazquezae* | WICH | EtOH (-80) | MEXICO: Puebla. Cuetzalan del progreso, Cascada velo de novia. N 20º 0’ 20’’ W 97º 2’ 31’’. 157 msnm. VII-24-2012. Col: Reyes, Bexa, Villerias, and Jimenez | KY671343 | KY671381 | KY671417 |
| CB028 | *Odontotaenius striatopunctatus* | WICH | EtOH (-80) | MEXICO: Puebla. Cuetzalan del progreso, delante de Iztahuaca. N 20º 1’ 19.6’’ W 97º 30’ 10.6’’. 756 msnm. VII-24-2012. Col: Reyes, Bexa, Villerias, and Jimenez | KY671344 | KY671382 | KY671418 |
| CB029 | *Chondrocephalus purulensis* | WICH | EtOH (-80) | GUATEMALA: Alta Verapaz. Chamil. | KY671345 | KY671383 | KY671419 |
| CB035 | *Yumtaax veracrucensis* | INECOL | Pinned | MEXICO: Veracruz. Congr. La Guacamaya, Mpio. Chiconquiaco. Altitud. 1950 m. 19°45'51.4"N 96°48'1.7"W. Encinar perturbado En tronco podrido 6-Octobre-2008. Col: P. Rojas | KY671346 | KY671384 | KY671421 |
| CB036 | *Yumtaax laticornis* | INECOL | Pinned | MEXICO: Veracruz. Calcahualoo Tecoanapa. Bosque mesofilo, Alt. 2,200 m. VI-1992. Col: Capistran y Delgado. | KY671347 | KY671385 | KY671420 |
| CB038 | *Petrejoides jalapensis* | INECOL | Pinned | MEXICO: Oaxaca. 10 Km S. Sn. Jeronimo Coatlan. 16°12.917'N 96°54.206'W 25-Mayo-2006. Alt. 2160 msnm. Col: Frankie, Montano, A. Valdez, Santibanez, Ballesteros | KY671348 | KY671386 | KY671422 |
| CB039 | *Petrejoides jalapensis* | INECOL | Pinned | MEXICO: Oaxaca. 10 Km S. Sn. Jeronimo Coatlan. 16°12.917'N 96°54.206'W 25-Mayo-2006. Alt. 2160 msnm. Col: Frankie, Montano, A. Valdez, Santibanez, Ballesteros | KY671349 | KY671387 | KY671423 |
| CB040 | *Petrejoides tenuis* | ALGI | Pinned | PANAMA: Chiriqui Prov. 2 k. NE Jurutungo, el. 1900 m. 21-Dec-1992. Col: A.R. Gillogly. | KY671350 | KY671388 | KY671424 |
| CB041 | *Petrejoides tenuis* | ALGI | Pinned | PANAMA: Chiriqui Prov. 2 k. NE Jurutungo, el. 1900 m. 21-Dec-1992. Col: A.R. Gillogly. | KY671351 | KY671389 |  |
| CB042 | *Petrejoides tenuis* | ALGI | Pinned | PANAMA: Chiriqui Prov. 2 k. NE Jurutungo, el. 1900 m. 21-Dec-1992. Col: A.R. Gillogly. | KY671352 | KY671390 |  |
| CB048 | *Spurius bicornis* | ALGI | Pinned | MEXICO: Chiapas. Mi. 18, new Tuxtla G./Acayucan Rd. 850 m. 12-X-1997. Col: Gillogly and Marshall. | KY671353 | KY671391 |  |
| CB049 | *Spurius* sp. | ALGI | Pinned | PANAMA: Chiriqui. Rsva. Fortuna, 3 de Nov. Trl. El. 1235-1350 m., 28-IV-2009. N8°45.768' W82°15.358' Col: Gillogly and Ward Jr. | KY671354 | KY671392 | KY671425 |
| CB050 | *Spurius* sp. | ALGI | Pinned | PANAMA: Chiriqui. Rsva. Fortuna, Samudio Trl. El. 1090 m. 27-IV-2009. N 8°44.1' W 82°14.8' Col: Gillogly and Ward Jr. | KY671355 | KY671393 | KY671426 |
| CB051 | *Popillius erotylus* | ALGI | Pinned | PANAMA: Bocas del Toro Pr. Km 8.8, Rambala-Almirante Rd., Rio La Gloria, el. 35 m. N 8°59'04" W 82°13'57". 8-I-2001. Col: Gillogly and Godwin | KY671356 |  | KY671427 |
| CB052 | *Popillius erotylus* | ALGI | Pinned | PANAMA: Bocas del Toro Pr. Km 8.8, Rambala-Almirante Rd., Rio La Gloria, el. 35 m. N 8°59'04" W 82°13'57". 8-I-2001. Col: Gillogly and Godwin | KY671357 |  | KY671428 |
| CB057 | *Chondrocephalus salvadorae* | UNSM | EtOH (-80) | EL SALVADOR: Santa Ana. Parq. Nac. Montecristo. N14°24'33" W89°22'20". 2175 m. VI-20-21-2002. Col: Ratcliffe et al. | KY671358 | KY671394 | KY671429 |
