# Supplementary figures and images for: Phylogeny of the genus Yumtaax Boucher (Coleoptera, Passalidae, Proculini): Taxonomic and evolutionary implications with descriptions of three new species
Source: Zookeys. 2017 Apr 10;(667):95–129. doi: 10.3897/zookeys.667.10716 (PMC5523388; doi:10.3897/zookeys.667.10716)

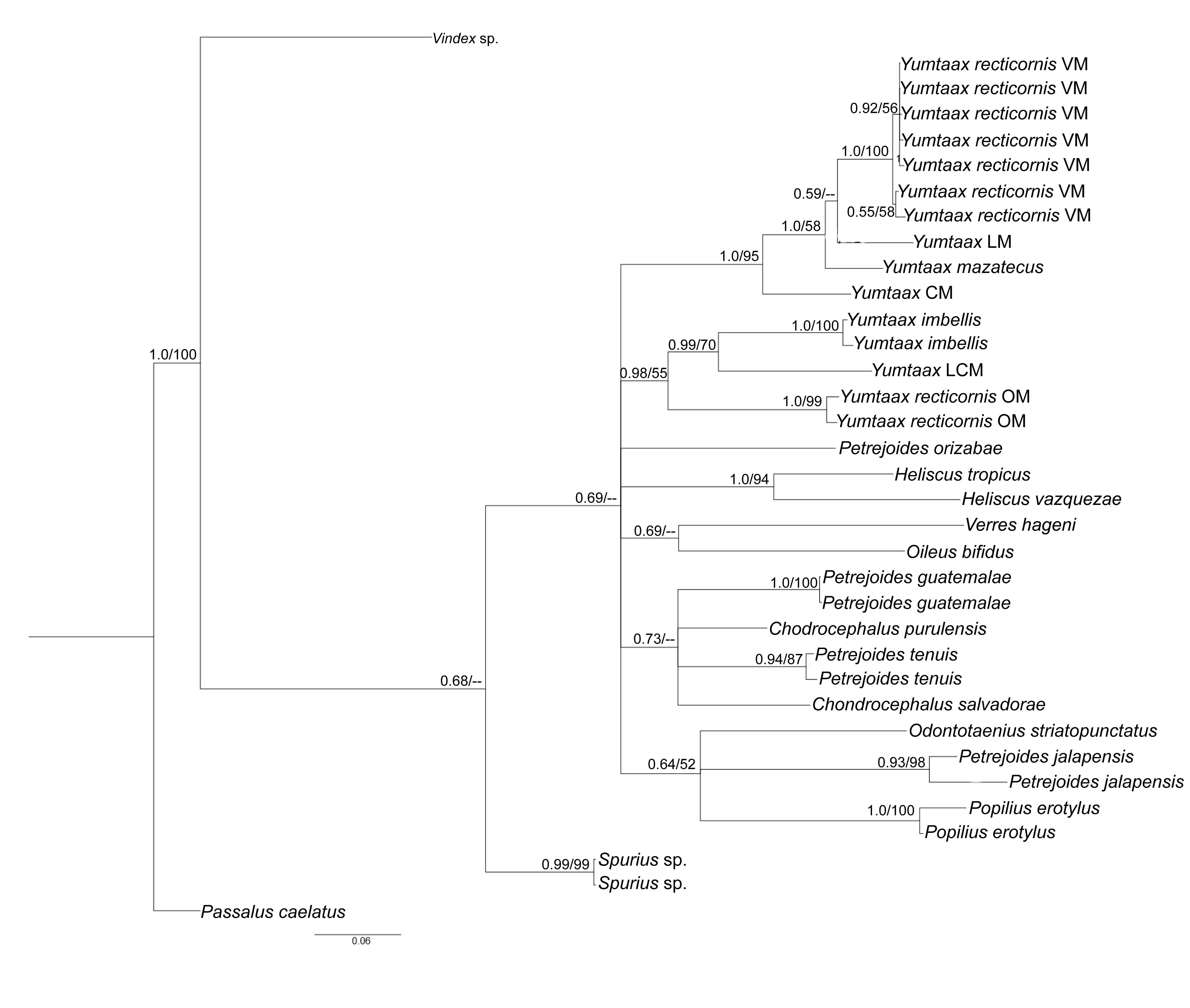

Supplement: Supplementary material 2 — Figure S1 [file zookeys-667-095-s002.jpg]

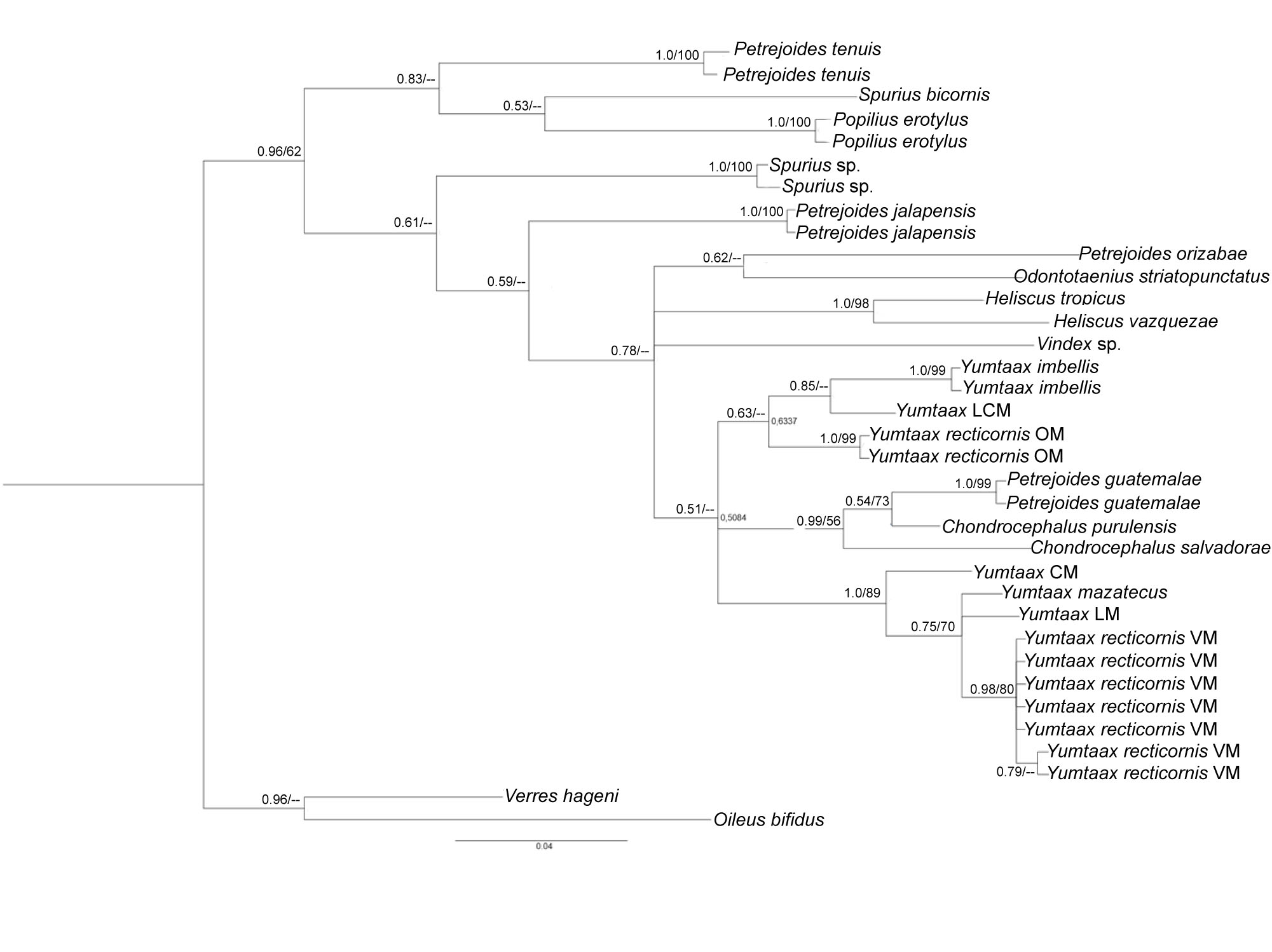

Supplement: Supplementary material 3 — Figure S2 [file zookeys-667-095-s003.jpg]

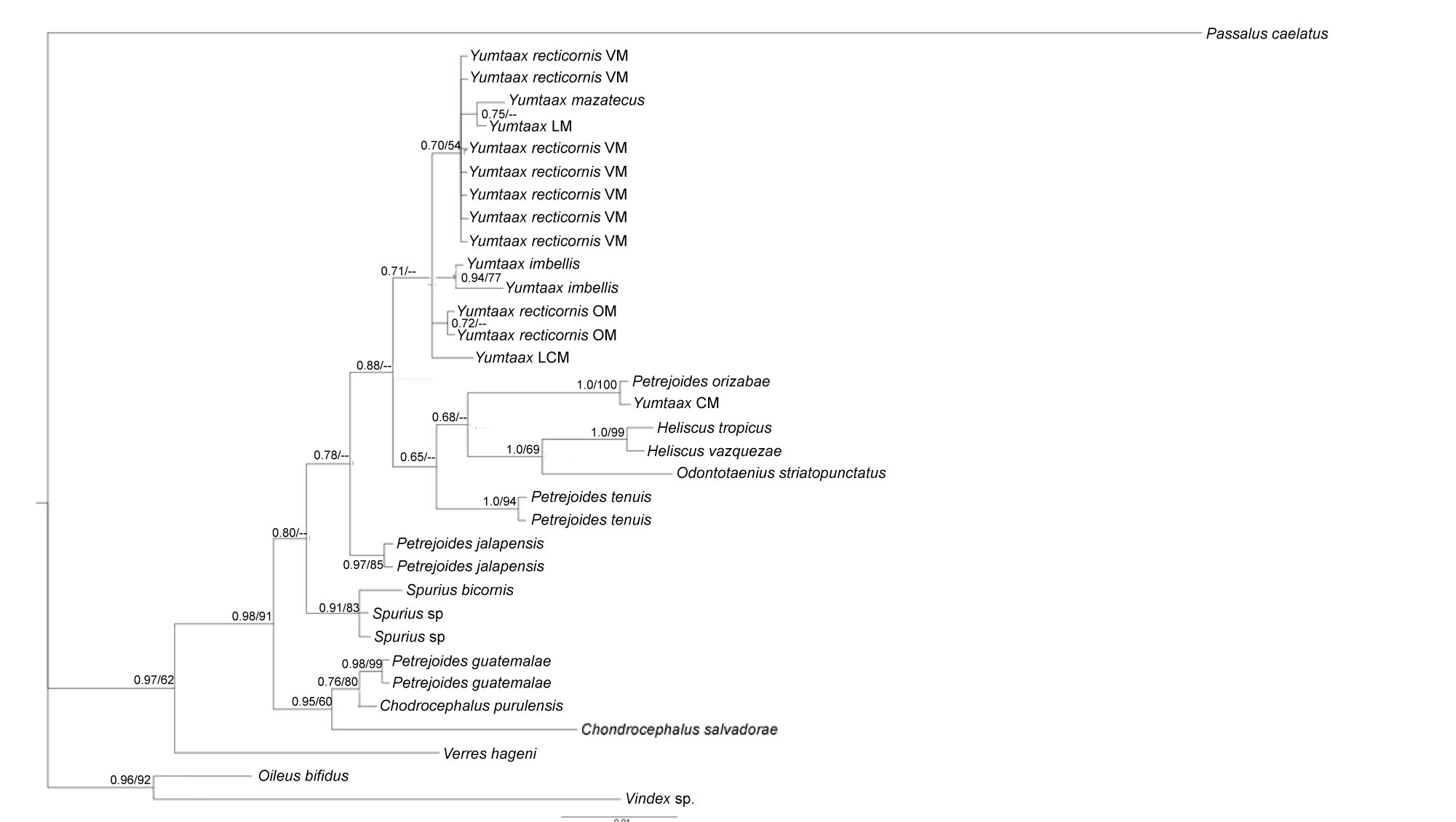

Supplement: Supplementary material 4 — Figure S3 [file zookeys-667-095-s004.jpg]
